# Discovery of oligodendrocyte enhancers that regulate *Sox10* expression

**DOI:** 10.1371/journal.pgen.1011778

**Published:** 2025-07-11

**Authors:** Hongjoo An, Chuandong Fan, Dongkyeong Kim, Huy Bui, Yungki Park

**Affiliations:** Institute for Myelin and Glia Exploration, Department of Biochemistry, Jacobs School of Medicine and Biomedical Sciences, State University of New York at Buffalo, Buffalo, New York, United States of America; Johns Hopkins University School of Medicine, UNITED STATES OF AMERICA

## Abstract

Oligodendrocytes (OLs) assemble myelin sheaths around axons in central nervous system (CNS). Myelin is essential for the saltatory conduction of action potentials and also performs other critical functions for the operation of the CNS. Sox10 (SRY-box containing gene 10) is a high-mobility group transcription factor that orchestrates the development of OLs. Despite its key role in OL biology, there is scant information on how the expression of *Sox10* is regulated in OL lineage cells. Especially, OL enhancers that control its transcription remain elusive. We have recently developed an innovative method that rationally links OL enhancers to target genes. This study applied the new method to *Sox10*, uncovering two OL enhancers for it (termed Sox10-E1 and Sox10-E2). Epigenome editing analysis revealed that Sox10-E1 and Sox10-E2 regulate *Sox10* expression non-redundantly. Luciferase assay and human and mouse brain multi-omics data show that, during the differentiation of OL precursor cells (OPCs) into OLs, the enhancer activity of Sox10-E1 does not change while that of Sox10-E2 decreases significantly. Chromatin interaction data indicate that the two *Sox10* enhancers lie close to the border of the *Sox10* topologically associating domain (TAD). Consistently, *Pick1*, a gene that is near the *Sox10* TAD border, is also under the transcriptional control of Sox10-E1 and Sox10-E2. Hence, genomic deletions involving Sox10-E1 and Sox10-E2 would perturb not only *SOX10*, but also *PICK1* and other genes, and may cause a pathology that is more complex than that of conventional Waardenburg-Shah syndrome that results from *SOX10* coding mutations.

## Author summary

Oligodendrocytes are a special cell type in brain and spinal cord and form myelin sheaths around axons. Myelin sheaths are lipid-rich structures that are essential for the rapid transmission of electrical impulses and also support the functional integrity of axons. Oligodendrocytes arise from neural stem cells. Sox10 is a transcription factor that plays an important role in the development of oligodendrocytes. Despite this key role, it remains poorly understood how the expression of *Sox10* is regulated in oligodendrocytes. In this study, we identified two DNA regulatory elements that govern the expression of *Sox10*. A comprehensive computational analysis uncovered five candidate regulatory elements for *Sox10*, and they were tested by CRISPRi, a cutting-edge epigenome editing tool. This investigation revealed two elements that are required for the expression of *Sox10* in oligodendrocytes. We also found that the two elements regulate the expression of *Pick1*, a gene next to *Sox10*, as well. This suggests that Waardenburg-Shah syndrome caused by the deletion of the two elements may exhibit a more complex pathology than conventional Waardenburg-Shah syndrome that arises from *SOX10* coding mutations because the former involves the loss-of-function of the two genes and their potential genetic interaction.

## Introduction

In central nervous system (CNS), oligodendrocytes (OLs) assemble myelin sheaths by wrapping their plasma membranes around axons [1]. Myelin is essential for the operation of the CNS. While the classical function of myelin is to accelerate the propagation of action potentials [2], recent research has uncovered additional functions. First, myelin ensures the integrity of axons [3–5]. Second, myelination is a dynamic process that can be modulated in response to environmental cues and experiences. This so-called adaptive myelination underpins learning and memory [6–10]. Third, myelin promotes synaptogenesis [11], contributing to the proper wiring of neural circuits. Fourth, myelin mediates the effects of social experience on animal behaviour [12–14]. These new discoveries highlight the dynamic and multifaceted nature of myelin in neural development and function.

Sox10 (SRY-box containing gene 10) is a high-mobility group transcription factor that orchestrates the development of OLs [15,16]. The expression of *Sox10* is turned on in OL precursor cells (OPCs) once they are specified from neural progenitors. Deletion of *Sox10* in OL lineage cells does not affect the survival and proliferation of OPCs [16]. Strikingly, however, their differentiation into myelin-forming OLs is completely blocked [15,16], resulting in lethal dysmyelination. Despite its crucial role in OLs, little is known about how the expression of *Sox10* is regulated in the OL lineage [17,18]. The expression of a gene is governed by upstream regulators acting on its enhancers (*cis*-regulatory DNA elements) [19]. To understand *Sox10* expression, thus, one needs to uncover its enhancers and transcription factors acting on them. Logically, the identification of enhancers would come first because, without the knowledge of enhancers, it would not be straightforward to identify transcription factors acting on them.

A puzzling feature of enhancers is that they can be found anywhere with regard to target genes [20]. This made it difficult to find *Sox10* enhancers. The traditional approach to this problem was to find conserved non-coding sequences in the vicinity of *Sox10* and test whether they work as enhancers in reporter assays [17,18]. If they do, *Sox10* is assumed to be under their control. Although this was the best one could do at the time, it had several limitations. First, one had to make an arbitrary decision about where and how far to look in search of conserved non-coding sequences. Second, even after the identification of enhancers, it was difficult to ascertain that they regulate *Sox10* expression because there was no tool available to modulate the activity of enhancers. Third, a single enhancer can control multiple genes [21]. Alternatively, a single gene may be regulated by multiple enhancers [22]. Upon the identification of enhancers, thus, it is critical to address their target gene specificity and functional relationship. Apparently, these were beyond the reach of the traditional approach. Due to these issues, progress has been slow in unveiling OL enhancers for *Sox10* [17,18,23].

Over the past decade, several breakthroughs have been made in genome research. First, we now know that enhancers and target genes tend to be found in the same topological associating domain (TAD), which is the fundamental unit of genome organization and function [24,25]. Second, enhancers are often marked with H3K27ac (acetylation of histone 3 lysine 27) [26]. These enable one to rationally narrow down the *Sox10* enhancer search space from the entire genome to a few discrete loci. Third, it is now possible to modulate the activity of enhancers *in situ* with epigenome editing tools such as CRISPRi [21,22,27–29], allowing one to directly determine whether candidate enhancers regulate the expression of *Sox10*. We took advantage of these developments to tackle the OL enhancer issue [30]. It has successfully uncovered enhancers for key OL genes such as *Myrf* [30], *Rgcc* [31], *Plp1* [32], *Olig2* [33], and *Cnp* [34]. Here, we applied it to *Sox10*, identifying two *Sox10* OL enhancers (termed Sox10-E1 and Sox10-E2). These two enhancers and the genomic architecture around *Sox10*, as revealed by Hi-C data, shed light on the OL regulation of *Sox10* expression and the pathology of Waardenburg-Shah syndrome that results from genomic deletions involving Sox10-E1 and Sox10-E2.

## Results

### A principled method to find OL enhancers for *Sox10*

**Fig 1:**
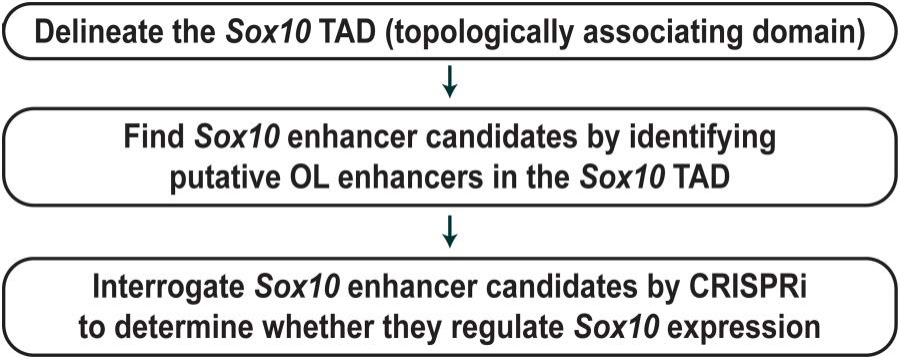
A principled method to find OL enhancers for *Sox10.*

An overview of our new enhancer-mapping method is given in Fig 1. In the first step, we analyze public chromatin interaction data to delineate the *Sox10* TAD. With the knowledge of the *Sox10* TAD, one can rationally narrow down the *Sox10* enhancer search space. Of note, the internal detail of a TAD differs between cell types because it reflects cell type-specific gene-enhancer interactions. In contrast, the boundary of a TAD tends to be conserved between cell types and species [24,35]. The latter property enabled us to define TADs for OL genes from non-OL Hi-C data [30–34]. Recently, Hi-C data for human OLs became available [36], facilitating the TAD analysis of OL genes. In the second step (Fig 1), we identify all putative OL enhancers in the *Sox10* TAD, which are qualified to be *Sox10* enhancer candidates because they reside in the same TAD as *Sox10*. Our previous study generated a genome-wide map of putative OL enhancers by integrating public OL ChIP-seq data [30]. This map is compared with the *Sox10* TAD to uncover putative OL enhancers in it. In essence, the first and second steps reduce the enhancer search space from the entire genome to a few discrete loci. In the last step (Fig 1), we inactivate *Sox10* enhancer candidates with CRISPRi to determine whether they regulate *Sox10* expression. CRISPRi is a cutting-edge epigenome editing technique that can silence enhancers and promoters in the genomic context [21,22,27–29]. If the region targeted by CRISPRi happens to be an *Sox10* enhancer, its CRISPRi silencing would downregulate *Sox10* expression. This is how one can identify *Sox10* enhancers by CRISPRi.

### TAD analysis for *Sox10*

**Fig 2:**
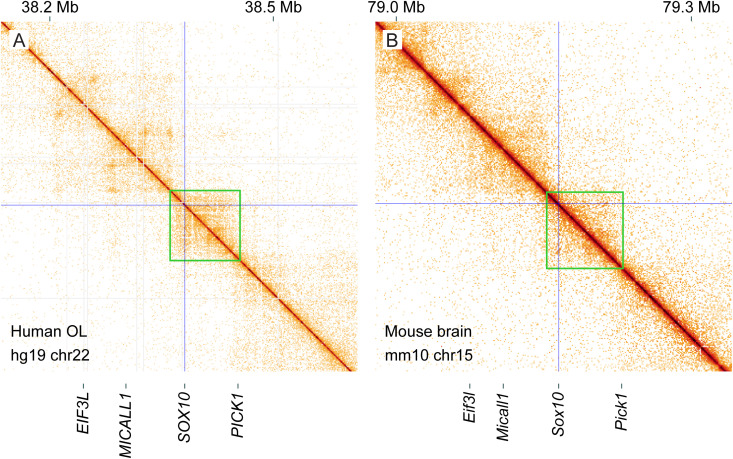
TAD analysis for *Sox10.* (A) Human OL Hi-C data. (B) Mouse brain Hi-C data. In both panels, the genome is on the diagonal. Off the diagonal, the interaction strength between two loci is indicated by color tone. White means no interaction, and orange indicates a strong interaction. The *SOX10*/*Sox10* promoter locations are marked by thin crossing lines. The *SOX10*/*Sox10* TAD is marked by a green box in each panel. Please see Materials and Methods for the sources of these data. These Figs were generated by HiGlass [84].

To delineate the *Sox10* TAD, we analyzed human and mouse Hi-C data [36,37]. In each panel of Fig 2, the diagonal represents the genome. Off the diagonal, the interaction strength between two loci is indicated by color. Orange signifies a strong interaction while white means no interaction. The positions of the *SOX10*/*Sox10* promoters are marked by thin blue lines. The Hi-C data for human OLs shows that *SOX10* resides in a TAD that spans about 100 kb (marked by a green box in Fig 2A). To see if this TAD is conserved in mice, we examined Hi-C data for the mouse brain (Fig 2B). It reveals that the *Sox10* TAD (marked by a green box) is essentially the same as the human one, as judged by the locations of the TAD boundary and nearby genes. The *Sox10* TAD spans about 80 kb, which makes sense because the mouse genome is smaller than the human one. Also, the TAD boundary is clearer in the human Hi-C data because it is from a single cell type (OLs) while the mouse data is from a complex tissue (brain). In light of the strong evolutionary conservation of the *Sox10* TAD, critical OL enhancers for *Sox10* are likely to be found in it.

### Identification of five *Sox10* enhancer candidates

**Fig 3:**
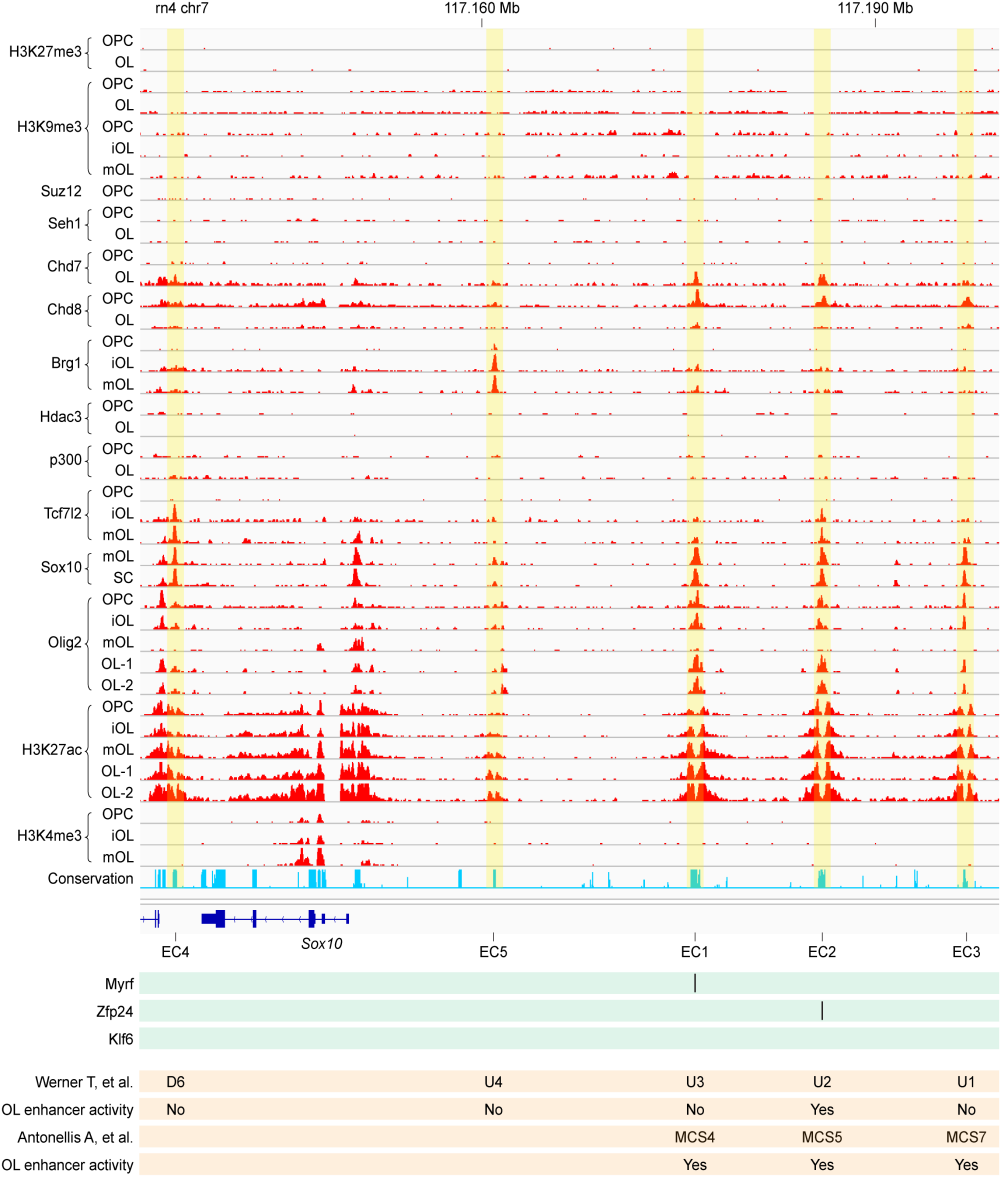
Five *Sox10* ECs. Rat OL ChIP-seq data were compiled for the five Sox10 ECs. Also shown are the locations of non-coding sequences examined by the two large-scale reporter assays [17,18] and their match with the five Sox10 ECs. iOL: immature OL. mOL: mature OL. SC: spinal cord. For the Myrf ChIP-seq data, only peak locations are shown because the raw data is not available. The mouse Zfp24 and Klf6 ChIP-seq data were mapped to the rat genome by LiftOver. OL^#^ and OL*: OLs treated with vehicle and lysophosphatidylcholine, respectively. Please see Materials and Methods for the sources of these data. This Fig was generated by the IGV browser [85].

We compared our genome-wide map of putative OL enhancers [30] with the *Sox10* TAD. This comparison unveiled five putative OL enhancers in the *Sox10* TAD, referred to as *Sox10* enhancer candidates (EC1–5 in Fig 3). The ranking was based on the strength of the underlying data, with EC1 being the best EC and EC5 being the worst. They are all decorated by an H3K27ac peak-valley-peak, an epigenetic hallmark associated with active enhancers [26]. They do not overlap with peaks of H3K4me3 (promoter), H3K9me3 (constitutive heterochromatin), and H3K27me3 (facultative heterochromatin). Key OL transcription factors such as Olig2, Sox10, Tcf7l2, Zfp24, and Myrf bind to them, suggesting their potential role in OL-specific gene expression.

Conserved non-coding sequences around *Sox10* were extensively examined by reporter assays in an effort to find *Sox10* enhancers [17,18]. The overlap between our five ECs and the conserved sequences interrogated by the two large-scale studies [17,18] is summarized in Fig 3. Also shown are the reporter assay results from the two studies.

### CRISPRi interrogation of the five *Sox10* enhancer candidates

CRISPRi is a cutting-edge epigenome editing technique [21,22,27–29]. In CRISPRi, dCas9-KRAB, a fusion protein between a nuclease-null Cas9 (dCas9) and a KRAB domain, is delivered to a target locus by guide RNAs (gRNAs). dCas9-KRAB silences the target locus by inducing trimethylation of H3K9 (K9 of histone 3) [29]. If the target locus is an enhancer, its CRISPRi silencing would decrease the expression of target genes.

To silence the five *Sox10* ECs, dCas9-KRAB was delivered to each by four independent gRNAs (G1-4) in Oli-neu cells, a widely used OL cell line [38]. The four gRNAs (G1, G2, G3, and G4) are different gRNAs targeting slightly different locations in each EC. gRNAs were cloned into an in-house piggyBac-based plasmid and inserted into the genome of Oli-neu cells that express dCas9-KRAB in a doxycycline-dependent manner (S1 Fig). In resulting cell lines, the expression of gRNAs was constitutive while that of dCas9-KRAB was induced by doxycycline. In parallel, four control cell lines were generated. In two of them, scrambled gRNAs called Scr1 and Scr2 were inserted into the genome. These scrambled gRNAs do not have a specific target in the mouse genome and were used as negative controls to assess the effects of the experimental manipulation. In the other two, two gRNAs that deliver dCas9-KRAB to the *Sox10* promoter (Pro1 and Pro2) were inserted into the genome to be used as positive controls. Please note that we generated these cell lines by a transposon-assisted approach (see Materials and Methods), which overcomes the limitations of the conventional random insertion-based method.

**Fig 4:**
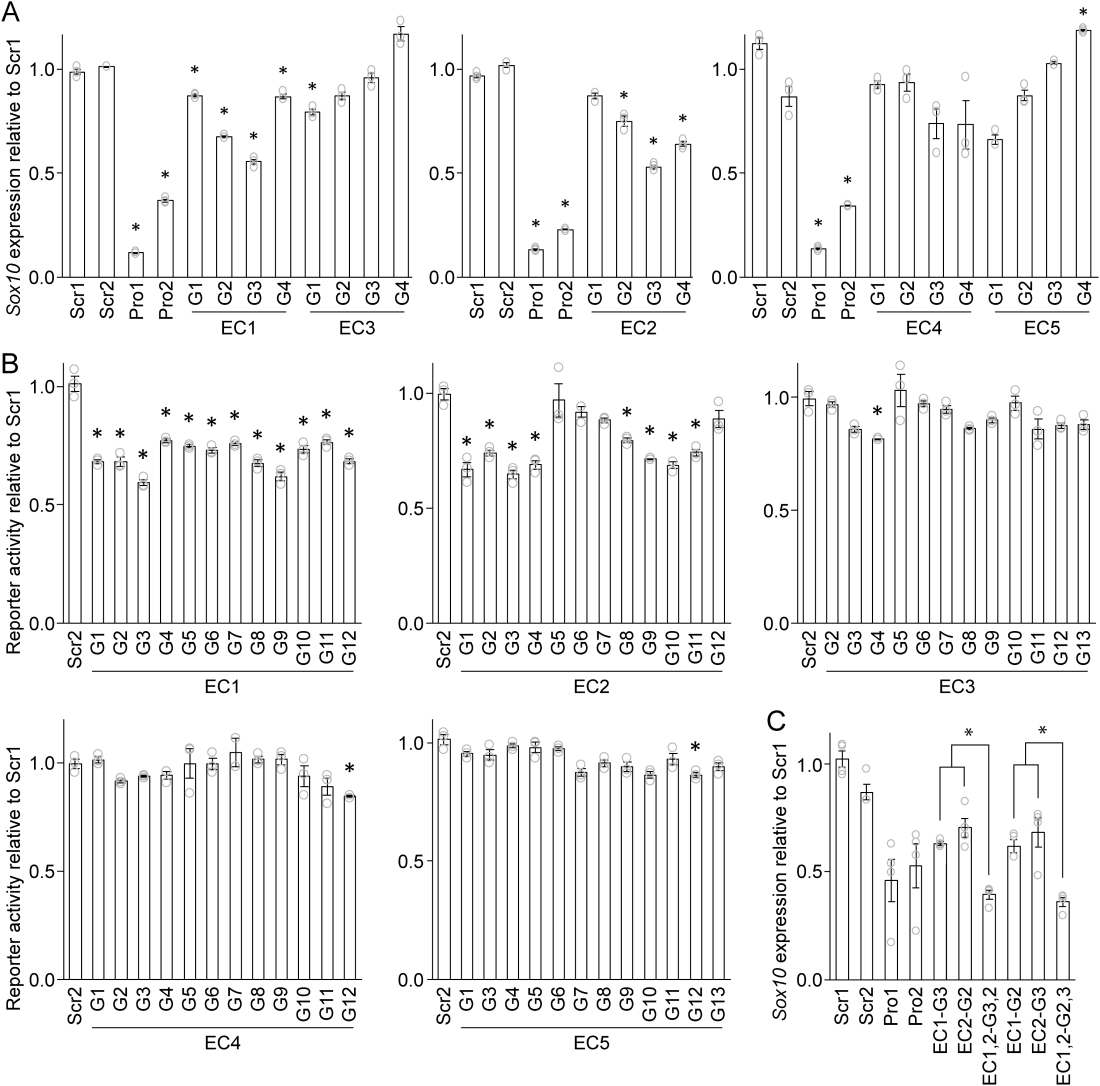
CRISPRi interrogation of the *Sox10* ECs. (A) RT-qPCR analysis of *Sox10* expression in Oli-neu cells after CRISPRi knockdown of the *Sox10* ECs. G1-4 indicate different gRNAs targeting slightly different locations in each EC. Shown are data points and their mean and standard error. *p < 3.33 × 10^-2^ by Student’s *t t*est for comparison with Scr1. (B) Luciferase assay for the *Sox10* ECs. G1-4 for each EC are the same as those in panel A, and the other gRNAs are additionally generated for the luciferase assay. Shown are data points and their mean and standard error. *p < 4.80 × 10^-2^ by Student’s *t* tes*t* for comparison with Scr1. (C) Effect of simultaneously silencing EC1 and EC2 on the expression of *Sox10* in Oli-neu cells. *p < 7.37 × 10^-3^ by Student’s *t* test. The raw da*t*a for Fig 4 are available in S1 Data.

As expected, the expression of *Sox10* went down when dCas9-KRAB was brought to its promoter (Pro1 and Pro2 compared to Scr1, Fig 4A), validating our experimental system. The expression of *Sox10* also dropped significantly when EC1 or EC2 was silenced by CRISPRi (Fig 4A). For example, all four gRNAs for EC1 (G1-4) and three of the four gRNAs for EC2 (G2-4) led to the downregulation of *Sox10*. In contrast, there was no consistent change in *Sox10* expression when dCas9-KRAB was targeted at the other three ECs. To corroborate these results, we retested the five ECs with more gRNAs in a luciferase assay that utilizes ECR9, an OL enhancer that sensitively responds to Sox10 [16]. In this experiment, dCas9-KRAB was targeted at the native EC loci, and the expression level of *Sox10* was read out by ECR9. When dCas9-KRAB was brought to EC1 in the genome by 12 different gRNAs, the reporter activity of ECR9 decreased significantly for all 12 gRNAs (G1-12, Fig 4B). The same was true for 8 of 12 gRNAs for EC2. In contrast, only 1 of 12 gRNAs came out positive for EC3, EC4, and EC5 (Fig 4B). These observations confirm that EC1 and EC2 regulate the expression of *Sox10* in Oli-neu cells. The negative results for EC4 and EC5, which are closer to the *Sox10* promoter than EC1 and EC2, rule out the possibility that CRISPRi repression non-specifically spreads from EC1 or EC2 to the *Sox10* promoter. This conclusion was further strengthened by a luciferase assay for three control genomic regions (S2 Fig).

To determine the functional relationship between EC1 and EC2, we silenced them together and compared its effect with that of individual enhancer inactivation. When EC1 and EC2 were simultaneously knocked down by CRISPRi, through either EC1-G3 and EC2-G2 or EC1-G2 and EC2-G3, the expression of *Sox10* went down to a greater extent compared to when individual enhancers were silenced (Fig 4C). These results indicate that EC1 and EC2 are not redundant.

Although Oli-neu cells mimic OL lineage cells in many aspects, they are not OL lineage cells. Key findings from Oli-neu cells have to be replicated with primary OLs to ensure physiological significance. To test EC1 and EC2 in primary OLs, we repeated the CRISPRi experiment with mouse OLs [39,40]. Challenges with transfection efficiency and the infeasibility of drug selection made it impossible to perform RT-qPCR. Instead, we resorted to quantitative immunofluorescence, as in our previous studies [30,32–34]. In this experiment, the effect of silencing an enhancer on gene expression is assessed in individual cells by quantitative immunofluorescence. Therefore, high transfection efficiency is not necessary. dCas9-KRAB and gRNA plasmids were co-transfected into mouse OPCs that were purified by immunopanning (see Materials and Methods). Transfected OPCs were cultured in the differentiation condition for 2 days to induce their differentiation into OLs. They were then stained for GFP (expressed by gRNA plasmids and identifying transfected cells) and Sox10. As above, Scr1 and Scr2 were used as negative control gRNAs.

**Fig 5:**
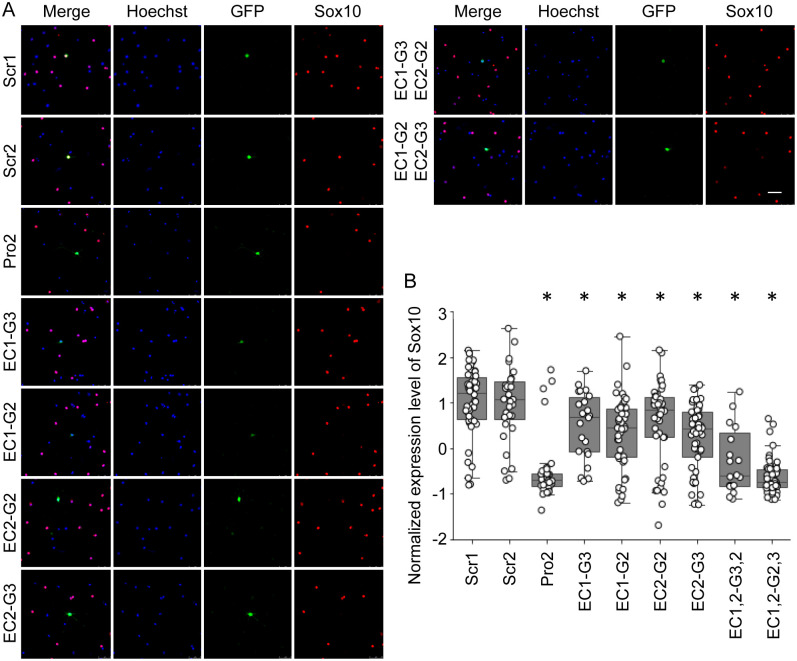
Quantitative immunofluorescence of Sox10 in mouse OLs after CRISPRi knockdown of EC1 and EC2. (A) Representative images of the 9 samples. Scale bar, 50 µm. (B) Boxplot of Sox10 signals for the 9 samples. Sox10 signals were quantified for individual cells by CellProfiler and compared among the samples. *p < 4.26 × 10^-2^ by Student’s *t t*est for comparison with Scr1. The raw data for Fig 5B are available in S2 Data.

For each of the 9 samples (Fig 5A), at least 50 pictures were taken because 50 pictures are usually enough to get robust results. Signals from three fluorescence channels (Hoechst, GFP, and Sox10) were quantified for individual OLs by CellProfiler [41]. The Sox10 expression levels of all cells in each picture were standardized (z-score normalization). The basic idea behind this z-score normalization is that the best controls for GFP+ cells are their untransfected neighbors. With the z scores of GFP+ cells, we compare their Sox10 expression levels with those of their untransfected neighbors while eliminating artifacts coming from staining and fluorescence microscope. In theory, the z scores for Scr1 and Scr2 should be 0. Due to the partial overlap of GFP and RFP spectra, however, their means tend to be greater than 0 (Fig 5B). When dCas9-KRAB was targeted at the *Sox10* promoter (Pro2), the Sox10 level went down substantially (compared to Scr1, Fig 5B), validating our experimental system.The same was also true when dCas9-KRAB was targeted at EC1, EC2, or EC1 and EC2. These results demonstrate that EC1 and EC2 promote *Sox10* expression in mouse OLs as well. To better reflect this regulatory relationship, EC1 and EC2 will henceforth be referred to as Sox10-E1 (Sox10 enhancer 1) and Sox10-E2 (Sox10 enhancer 2), respectively.

### Target gene specificity of Sox10-E1 and Sox10-E2

**Fig 6:**
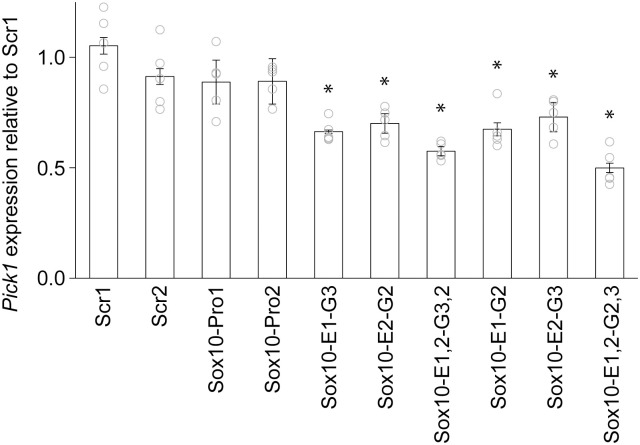
Effect of Sox10-E1 and Sox10-E2 on the expression of *Pick1.* Shown are data points and their mean and standard error. *p < 2.60 × 10^-2^ by Student’s *t t*est for comparison with Scr1. The raw data for Fig 6 are available in S3 Data.

It is not uncommon for enhancers to regulate multiple genes. *Pick1* is close to the border of the *Sox10* TAD (Fig 2), suggesting that it may also be under the transcriptional control of Sox10-E1 (EC1) and Sox10-E2 (EC2). To test this idea, we reanalyzed the RNA samples of Fig 4C by RT-qPCR. The expression of *Pick1* decreased when dCas9-KRAB was delivered to Sox10-E1, Sox10-E2, or Sox10-E1 and Sox10-E1 (Fig 6). In contrast, silencing the *Sox10* promoter did not affect *Pick1* expression. These results indicate that *Pick1* is also a target gene of Sox10-E1 and Sox10-E2 in Oli-neu cells, suggesting that the same may also be true in other cell types. This finding has a significant implication for the pathogenesis of SOX10-linked diseases (see Discussion).

### Cell type specificity of Sox10-E1 and Sox10-E2

**Fig 7:**
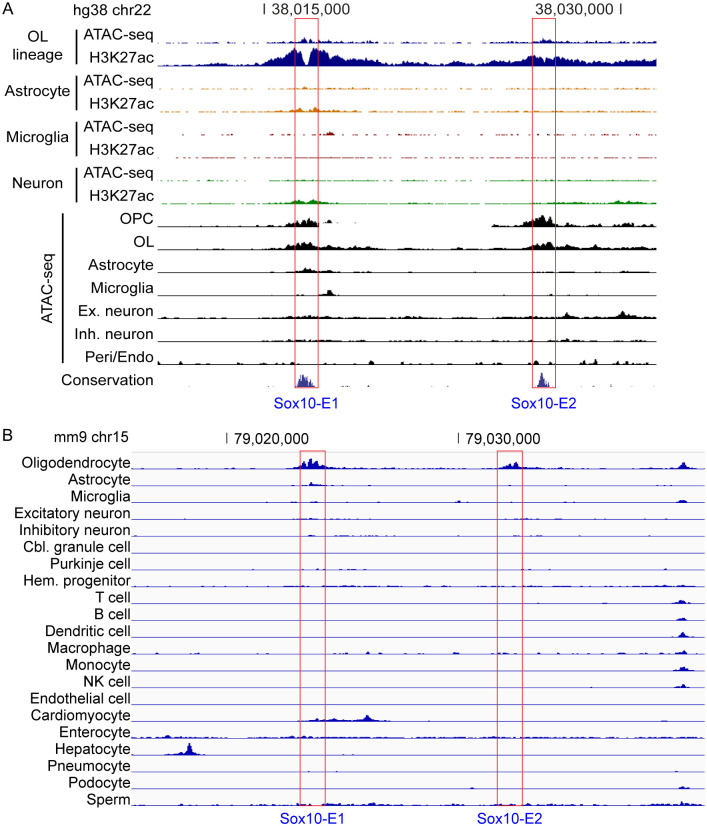
Cell type specificity of Sox10-E1 and Sox10-E2. (A) Human brain cell type-specific ChIP-seq and ATAC-seq data for Sox10-E1 and Sox10-E2. (B) Mouse single-cell ATAC-seq data for Sox10-E1 and Sox10-E2. Please see Materials and Methods for the sources of these data.

We surveyed public data to check the cell type specificity of Sox10-E1 and Sox10-E2. First, we inspected the human brain single-nucleus ATAC-seq data from Swarup and colleagues [42]. For an objective comparison of peaks among different cell types, the data were normalized by setting the peaks over *GAPDH* and *ACTB* to the same heights. The normalized data shows that Sox10-E1 and Sox10-E2 are open only in the OL lineage (OPC and OL, Fig 7A). Second, we looked up the human brain cell type-specific ChIP-seq and ATAC-seq data from Glass and co-workers [43]. These data were normalized in the same way. Sox10-E1 and Sox10-E2 coincide with OL-specific H3K27ac peak-valley-peaks and ATAC-seq peaks (Fig 7A), confirming that Sox10-E1 and Sox10-E2 are active only in the OL lineage in the human brain. Third, to check the specificity of Sox10-E1 and Sox10-E2 more broadly, we examined the H3K27ac ChIP-seq data from the NIH Roadmap Epigenomics Project [44], which were normalized in the same manner. It reveals that Sox10-E1 and Sox10-E2 are active mostly in the brain (S3 Fig). Finally, we checked the mouse single-cell ATAC-seq data from Shendure and colleagues, which were clustered into 21 cell types and normalized in the same way [45]. This dataset does not have data for OPCs. It indicates that Sox10-E1 and Sox10-E2 are specific to OLs (Fig 7B). Taken together, Sox10-E1 and Sox10-E2 seem specific to the OL lineage among the cell types and tissues examined by the public data. We note that Sox10-E1 and Sox10-E2 have been reported to be active in other cell lineages such as melanoma cells, neural crest cells, and peripheral neurons/glia [17,18,46–48], which are poorly represented by the public data.

### Temporal dynamics of Sox10-E1 and Sox10-E2

**Fig 8:**
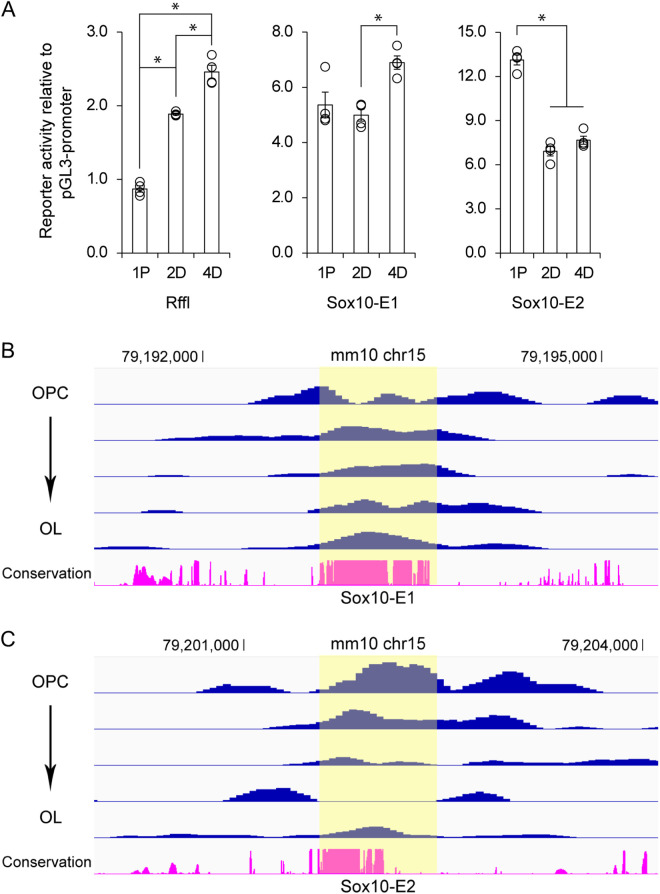
Temporal dynamics of Sox10-E1 and Sox10-E2 along the OL lineage. (A) Mouse OPCs transfected with reporters were cultured in the following conditions: 1 day of proliferation [1P], 2 days of differentiation [2D], and 4 days of differentiation [4D]. Reporter activity was determined by a luciferase assay. Shown are data points and their mean and standard error. *p < 5.58 × 10^-3^ by Student’s *t t*est. The raw data for Fig 8A are available in S4 Data. (B & C) The 256 OL lineage cells were ordered along the lineage by Monocle and clustered into 5 groups. The ATAC-seq data for each group was normalized for an objective comparison of peak heights. These Figs were generated by using the IGV browser. Please see Materials and Methods for the sources of these data.

*Sox10* is expressed throughout the OL lineage. Sox10-E1 and Sox10-E2 may play different roles in *Sox10* expression for OPCs and OLs. To test this hypothesis, the enhancer activity of Sox10-E1 and Sox10-E2 was examined by a luciferase assay in OPCs and OLs. Mouse OPCs transfected with reporters were cultured in the following conditions: 1 day of proliferation (1P), 2 days of differentiation (2D), and 4 days of differentiation (4D). Rffl, an OL-specific enhancer in the *Rffl* locus [49–51], was used as a control, as in our previous studies [30,31]. Successful differentiation of OPCs into OLs was confirmed by the increased reporter activity of Rffl (Fig 8A). Under these conditions, the reporter activity of Sox10-E1 (U3/MCS4) was about the same between OPCs and OLs while that of Sox10-E2 (U2/MCS5) significantly decreased during the differentiation process (Fig 8A). These observations suggest that Sox10-E1 is equally active in OPCs and OLs whereas Sox10-E2 is more active in OPCs than in OLs. This conclusion is corroborated by the human brain ATAC-seq data (Fig 7A), which shows that the Sox10-E1 peak does not change between OPCs and OLs while the Sox10-E2 peak is bigger in OPCs than in OLs.

To gain further support for our conclusion, we examined a public mouse brain ISSAAC-seq data [52]. ISSAAC-seq is a revolutionary method that determines the gene expression and chromatin accessibility of complex tissues simultaneously at the single cell level [52]. The public mouse brain ISSAAC-seq data consists of a single-cell RNA-seq data and a single-cell ATAC-seq data for the same brain cells. A clustering analysis of the single-cell RNA-seq data identified 256 OL lineage cells, which were ordered along the lineage by a pseudotime analysis (*i.e.*, from OPCs to OLs), as described before [34]. We then looked up the single-cell ATAC-seq data for the chromatin accessibility of Sox10-E1 and Sox10-E2 for the 256 OL lineage cells. The Sox10-E1 peak seems constant throughout the OL lineage (Fig 8B). In contrast, the Sox10-E2 peak appears to decrease during differentiation (Fig 8C). These results reinforce our conclusion about the temporal dynamics of Sox10-E1 and Sox10-E2.

## Discussion

Sox10 is a master regulator of OL development [15,16]. The pro-differentiation function of Sox10 depends on its proper expression, as evidenced by the delayed differentiation of OPCs into OLs upon the loss of one allele [53]. This expression requirement also holds true for other biological contexts [54–57]. All these point to the importance of understanding how the transcription of *Sox10* is regulated. The present study addresses it in the OL lineage. To elucidate the expression of *Sox10* in OLs, one needs to identify OL enhancers that govern it and upstream regulators acting on them. Logically, the first step would be the identification of OL enhancers for *Sox10*, because without such knowledge, it becomes challenging to determine the transcription factors that influence them.

Our analysis of the public Hi-C data narrowed down the *Sox10* enhancer search space from the entire genome to a rather small region of approximately 80 kb in the mouse genome. Remarkably, this region coincides with the regions that were extensively interrogated by the two large-scale reporter assays [17,18]. These studies were performed before the development of Hi-C techniques and the formulation of TADs as the fundamental unit of genome organization and function. Thus, they had to be arbitrary in their decisions regarding where and how far to look in the genome for *Sox10* enhancer candidates. Nonetheless, they were quite accurate in delineating the *Sox10* enhancer search space. Our genome-wide map of putative OL enhancers detected five putative OL enhancers in the *Sox10* TAD, which became *Sox10* EC1–5. Epigenome editing analysis revealed that Sox10-E1 (EC1) and Sox10-E2 (EC2) regulate *Sox10* expression non-redundantly. Our luciferase assay suggests that during the differentiation of OPCs into OLs, the enhancer activity of Sox10-E1 does not change while that of Sox10-E2 decreases significantly. This conclusion is supported by the human brain single-nucleus ATAC-seq data and the mouse brain ISSAAC-seq data. It also aligns with a previous report that Sox10-E2 (U2 in their nomenclature) is much more active in OPCs than in OLs [23].

*Pick1* lies close to the border of the *Sox10* TAD. TAD boundaries work as insulators that limit the influence of enhancers to those genes in the same TAD [24,25,58]. However, the degree of insulation is not the same for all boundaries [59]. We wondered whether *Pick1* is completely insulated from Sox10-E1 and Sox10-E2. When they were silenced by CRISPRi, the expression of *Pick1* decreased substantially in Oli-neu cells (Fig 6), suggesting that Sox10-E1 and Sox10-E2 may activate *Pick1* in other cell types. Mutations in non-coding sequences that are thought to regulate the expression of *SOX10* in neural crest-derived tissues have been associated with SOX10-related diseases [48,55–57]. Our finding that *Pick1* is also a target gene of Sox10-E1 and Sox10-E2 suggests that diseases caused by genomic deletions involving Sox10-E1 and Sox10-E2 may exhibit a more complex pathology than those resulting from *SOX10* coding mutations because of the combined loss of *SOX10* and *PICK1* and their potential genetic interactions. Further, if the deletions include the border of the *Sox10* TAD, the TAD architecture around *SOX10* and *PICK1* may be disrupted, derailing the expression of many more genes. In this case, an even more complex pathology may be expected.

Myrf, a master regulator of OL development [60], binds to Sox10-E1 [49] (Fig 3), suggesting that Myrf may activate the expression of *Sox10* via Sox10-E1. Scanning motif incidences with FIMO [61], we found one good match for the Myrf motif [50] in Sox10-E1 (S4A and S4B Fig). We found that Myrf binds to it (S4B Fig). Further, our luciferase assay indicated that it mediates the impact of Myrf on Sox10-E1 (S4C Fig). However, one caveat is that the Myrf ChIP-seq experiment and our luciferase assay were performed with overexpressed Myrf. To determine whether endogenous Myrf acts on the Myrf motif incidence in Sox10-E1, we performed a luciferase assay. The goal of this experiment was to compare the reporter activity of Sox10-E1 with that of the mutant version (S4D Fig), which does not respond to overexpressed Myrf and whose binding to Myrf is greatly decreased. Mouse OPCs transfected with various reporters, including the wild type and the mutant Sox10-E1, were cultured in the following conditions: 1 day of proliferation (1P), 2 days of differentiation (2D), and 4 days of differentiation (4D). If endogenous Myrf acts on the Myrf motif incidence in Sox10-E1, there would be a significant difference in activity between the wild type and the mutant Sox10-E1 in the 2D and/or 4D conditions, but not in the 1P condition. Successful differentiation of OPCs into OLs was confirmed by the increased reporter activity of Rffl, as in Fig 8A. Under these conditions, we failed to observe the expected pattern in the luciferase assay results (S4D Fig). Two-way analysis of variance (ANOVA) revealed that the mutation of the Myrf motif incidence lowers the reporter activity of Sox10-E1 in all three conditions. Thus, it remains unclear whether endogenous Myrf acts on Sox10-E1 to regulate *Sox10* expression.

Our study has two main limitations. First, although the CRISPRi results for Sox10-E1 and Sox10-E2 are solid, they must be validated *in vivo* by a deletion experiment. It would necessitate the generation of mice where each of the two enhancers is flanked by loxP sequences for cell type-specific deletion. We intend to conduct such a study in the future. Second, the primary CRISPRi screen was limited to Oli-neu cells. EC1 and EC2, which yielded positive results, were subsequently validated in primary cells. However, we may have missed ECs3–5 due to the exclusive use of Oli-neu cells. To our knowledge, Oli-neu is the only reliable mouse cell line representative of the OL lineage, which supports the rationale for our study design. Notably, EC3 - identified as negative in our CRISPRi screen - has previously been shown not to regulate *Sox10* expression in the CNS, further supporting the validity of our CRISPRi findings in Oli-neu cells.

## Materials and methods

### Ethics statement

All animal husbandry and experiments were performed following a protocol approved by the Institutional Animal Care and Use Committee (IACUC) of State University of New York at Buffalo (Protocol AR202300035).

### Animal procedures, tissue harvest, and cell culture

OPCs were purified from rat and mouse pups by immunopanning [39,40]. The original protocol for mouse OPCs [39] did not work in our hands. Thus, we made one significant change. Instead of a positive selection with anti-Pdgfrα antibody [39], we performed a negative selection with anti-O1 antibody and a positive selection with anti-O4 antibody, as described for rat OPCs [40]. Upon immunopanning, OPCs were amplified by culturing with PDGF-AA for 6–7 days. During this amplification period, non-OPCs are diluted out, resulting in a pure population of OPCs. A more detailed protocol is available upon request. OPCs and Oli-neu cells [38] were kept in a proliferative condition by supplementing the Sato media [40] with PDGF-AA (5 ng/mL), NT-3 (0.5 ng/mL), CNTF (5 ng/mL), and B-27 (1:200 dilution). To induce their differentiation, PDGF-AA was omitted, and T3 was added (40 ng/mL). For Oli-neu cells, PD 174265 (1 nM) was also added to inhibit EGFR tyrosine kinase. Cells were maintained in a humidified 7% CO_2_ incubator at 37°C. All transfections were carried out by using Lipofectamine 2000 as per the manufacturer’s instructions.

### CRISPRi constructs: dCas9-KRAB

Two dCas9-KRAB constructs were generated. First, “dCas9-KRAB-RB” (S1 Fig), a doxycycline-inducible dCas9-KRAB that can be integrated into the genome of Oli-neu cells, was generated by modifying pAAVS1-NDi-CRISPRi (Addgene 73497) as follows. First, an RB (RFP and blasticidin resistance) cassette was fused to the rtTA via P2A. Second, the inverted terminal repeats (ITRs) recognized by SB100X (Addgene 34879) [62] were inserted. dCas9-KRAB-RB was used to generate stable cell lines (see below). Second, “dCas9-KRAB-GP” was generated in the same way except that an GP (GFP and puromycin resistance) cassette was fused to the rtTA instead of the RB cassette. dCas9-KRAB-GP was used for quantitative immunofluorescence experiments. Sequence information for dCas9-KRAB-RB and dCas9-KRAB-GP was verified by Sanger sequencing.

### CRISPRi constructs: Guide RNAs

A guide RNA (gRNA)-expressing construct, “PB-GP-U6” (S1 Fig), was generated as follows. First, the content of PB-CA (Addgene 20960) was replaced by the sgRNA scaffold of lentiCRISPR v2 (Addgene 52961). Second, the GP cassette was inserted. gRNAs cloned into PB-GP-U6 were used to generate stable cell lines and for quantitative immunofluorescence experiments. Sequence information for all gRNA constructs was confirmed by Sanger sequencing. The sequences of the gRNAs used for the study are available in S1 Table.

### Stable cell line generation

dCas9-KRAB-RB and SB100X were co-transfected into Oli-neu cells [38]. SB100X [62], a hyperactive transposase, recognizes the ITRs of dCas9-KRAB-RB and integrates whatever flanked by the ITRs into the genome (S1 Fig). Hence, cells that proliferate in the presence of blasticidin express dCas9-KRAB in a doxycycline-dependent manner. Similarly, PB-GP-U6 and hypBase [63] were co-transfected into Oli-neu cells. hypBase, a hyperactive transposase, recognizes the ITRs of PB-GP-U6 and inserts whatever flanked by the ITRs into the genome (S1 Fig). Thus, cells that proliferate in the presence of puromycin constitutively express gRNAs. Of note, the SB100X and hypBase plasmids themselves are not inserted into the genome. They are diluted out during cell proliferation. The hypBase plasmid was generously provided by Breunig [64]. To generate stable cell lines for single gRNAs, four plasmids (dCas9-KRAB-RB, SB100X, PB-GP-U6, hypBase) were co-transfected, and transfected cells were subjected to drug selection with blasticidin and puromycin. The transposon-assisted genomic integration of plasmids is highly effective. Together with the fact that Oli-neu cells can easily be killed by blasticidin and puromycin, it allows us to produce desired cell lines in a week [30–33,65]. There is no need to grow single cell clones and check gene expression for them, as when generating stable cell lines via random genomic insertion. Oli-neu cells were cultured in the proliferation condition during the drug selection process. Once it is over, Oli-neu cells were treated with doxycycline (1 ug/ml) for 2 days to execute CRISPRi epigenome editing.

### RT-qPCR

Total RNA was purified by using Trizol (ThermoFisher 15596026), and cDNA was synthesized by the SuperScript First-Strand kit (Invitrogen 11904–018). Quantitative PCR was performed on C1000 Thermal Cycler with CFX96 optical reaction module (Bio-rad). *Gapdh* was used as a loading control. Each PCR reaction contained 2 µL of cDNA reverse transcribed from 1 ng of total RNA, 5 µL of the iTaq Universal SYBR Green Supermix (Bio-rad 1725124), and 500 nM of forward and reverse primers. The primer sequences are as follows.

*Gapdh* (forward): GGT GAA GGT CGG TGT GAA CGG

*Gapdh* (reverse): CTG GAA CAT GTA GAC CAT GTA GTT GAG G

*Sox10* (forward): GCA CGC AGA AAG CTA GCC G

*Sox10* (reverse): GAG CCT CTC AGC CTC CTC AAT G

*Pick1* (forward): GGG CCC AAT ACT GTC CTT GTC TC

*Pick1* (reverse): GCC ATT GAC CCC AGT GAT CTC

### Immunofluorescence

Cells were fixed with 4% formaldehyde and permeabilized with 0.1% Triton X-100. Upon blocking with 1% BSA, they were incubated with primary antibodies diluted in blocking buffer at 4°C overnight, followed by incubation with fluorochrome-conjugated secondary antibodies. Nuclei were stained with Hoechst 33342 (Invitrogen). Fluorescence was visualized with Leica DMi8 microscope with ORCA-Flash4.0 sCMOS camera. Reagents used for immunofluorescence are as follows: Sox10 (R&D SYSTEMS AF2864), EGFP (BioLegend 338001), donkey anti-Goat IgG, Alexa Fluor 594 (ThermoFisher A11058), and donkey anti-Rat IgG, Alexa Fluor 488 (ThermoFisher A21208).

### Luciferase assay

Sox10-E1 (mm10 chr15:79192912–79193720) and Sox10-E2 (mm10 chr15:79201371–79202190) were cloned into pGL3-promoter. Luciferase assay was performed by using the Firefly & Renilla Luciferase Single Tube Assay Kit from Biotium as per the manufacturer’s instructions. pRL-TK was used as an internal control. The ratio between firefly and renilla luciferase activities was taken as the reporter activity.

### OL ChIP-seq data

OL ChIP-seq data were downloaded from the Sequence Read Archive (SRA, https://www.ncbi.nlm.nih.gov/sra): GSE42454 (H3K9me3, Brg1, Olig2, H3K27ac, H3K4me3) [66], GSE72727 (Chd7, Sox10) [67], GSE119816 (Seh1) [68], GSE76411 (Hdac3, p300) [69], GSE82165 (Suz12) [70], GSE65119 (Tcf7l2) [71], GSE84011 (Olig2, H3K27ac) [72], GSE64703 (Sox10) [73], GSE107919 (Chd7, Chd8) [74], GSE101535 (Zfp24) [75], and GSE79243 (Klf6) [76]. The Myrf ChIP-seq data were downloaded from the journal website [49]. H3K27me3 and H3K9me3 data were kindly provided by Dr. Patrizia Casaccia [77]. ChIP-seq reads were mapped to rn4 by Bowtie2 [78], and peaks called by MACS2 [79].

### Public genomic data

The mouse brain Hi-C data [37] were downloaded from the 4DN Web Portal (https://4dnucleome.org). The human OL Hi-C data [36] were downloaded from a public box directory at https://github.com/dixonlab/scm3C-seq. Human brain single-nucleus ATAC-seq data were downloaded from the Swarup laboratory website [42]. Human brain cell type-specific ATAC-seq and ChIP-seq data from Glass and coworkers [43] are available at https://genome.ucsc.edu/s/nottalexi/glassLab_BrainCellTypes_hg19. The H3K27ac ChIP-seq data from the Roadmap Epigenomics Project [44] were visualized by the WASHU Epigenome Browser. Mouse single-cell ATAC-seq data [45] were downloaded from the Shendure laboratory website (https://atlas.brotmanbaty.org).

### Mouse brain ISSAAC-seq data

As described in the original paper [52], the single-cell RNA-seq reads were mapped to the mouse genome (mm10) by STAR [80]. The single-cell ATAC-seq reads were mapped to mm10 by Cell Ranger ATAC (10x Genomics). Mapped reads were analyzed by Seurat [81] and Signac [82]. Pseudotime analysis was carried out by Monocle [83]. Low-quality cells were excluded from the analysis – those with more than 1% of mitochondrial genes, those with less than 2% of transcriptional start site enrichment, those with greater than 4% of nucleosome signal patterns, and those with greater than 5% of blacklist region mapping. Altogether, 256 OL lineage cells were analyzed.

## Supporting information

S1 Fig Transposon-based plasmids used to generate stable cell lines. (PDF)

S2 Fig CRISPRi analysis of three control regions. (A) dCas9-KRAB was delivered to NC1, a region 1 kb upstream of the *Sox10* promoter, by 8 different gRNAs. The expression level of *Sox10* was measured by a luciferase assay with ECR9. (Left) The locations of the 8 gRNAs. (Right) The luciferase assay results. Pro2 was used as a positive control. Shown are data points and their mean and standard error. *p < 6.91 × 10^-4^ by Student’s *t* test. None of the 8 gRNAs affected the transcription of *Sox10*. (B) dCas9-KRAB was delivered to NC2, a region between the *Sox10* promoter and EC1, by 12 different gRNAs. The expression level of *Sox10* was measured by a luciferase assay with ECR9. (Left) The locations of the 12 gRNAs. G6, which is between G1 and G4, is not shown because of a low BLAT mapping score. (Right) The luciferase assay results. Pro2 was used as a positive control. Shown are data points and their mean and standard error. *p < 2.03 × 10^-3^ by Student’s *t* test. None of the 12 gRNAs affected the transcription of *Sox10*. (C) dCas9-KRAB was delivered to NC3, a region between EC1 and EC2, by 12 different gRNAs. The expression level of *Sox10* was measured by a luciferase assay with ECR9. (Left) The locations of the 12 gRNAs. G9, which is between G6 and G7, is not shown because of a low BLAT mapping score. (Right) The luciferase assay results. Pro2 was used as a positive control. Shown are data points and their mean and standard error. *p < 9.77 × 10^-3^ by Student’s *t* test. Promoter contact is not a privilege limited to enhancers. Non-enhancer regions can also contact the promoter of a gene. Though rare, some loci between EC1 and EC2 may contact the *Sox10* promoter and be open enough. If dCas9-KRAB is targeted at such a locus, the expression of *Sox10* would be downregulated, even though the targeted locus is not an enhancer. This phenomenon has previously been reported for a different gene (*Science* 2016 354:769). Of the 12 gRNAs, 3 led to the downregulation of *Sox10*. Notably, all three gRNAs map to the 3’ end of the region, arguing against the idea that CRISPRi repression non-specifically spreads from the target site to the *Sox10* promoter. Rather, these results suggest that the 3’ end of the region contacts the *Sox10* promoter and is open enough. The raw data for S2 Fig are available in S5 Data.
(PDF)

S3 Fig The NIH Roadmap Epigenomics Project H3K27ac ChIP-seq data for Sox10-E1 and Sox10-E2. (PDF)

S4 Fig Overexpressed Myrf, but not endogenous Myrf, acts on Sox10-E1. (A) The Myrf motif that mediates the sequence-specific DNA binding of Myrf. (B) DNA pulldown assay for Myrf and Sox10-E1. FLAG-Myrf (Myrf with an N-terminal FLAG tag) was expressed in Oli-neu cells, and cell lysates were mixed with either bare beads or beads coated with duplex DNA oligos (Sox10-E1-WT and Sox10-E1-MU). The duplex oligos contained either the wild type motif incidence (Sox10-E1-WT) or a mutated one (Sox10-E1-MU). The mixtures were separated into the sup and bead fractions by centrifuge, and both fractions were probed by FLAG antibodies. Immunoblotting showed that Myrf specifically bound Sox10-E1-WT and that this binding became weaker when the motif incidence was mutated (the bead fractions). The sup fraction revealed that comparable amounts of proteins were used for the three binding reactions, ruling out the trivial possibility that the specific binding of Myrf to Sox10-E1-WT is due to unequal protein amounts used for the binding reactions. IB: immunoblotting. (C) To test the functional significance of the Myrf motif incidence in Sox10-E1, we performed a luciferase assay in Oli-neu cells. The wild type and the mutant versions of Sox10-E1 were cloned into pGL3-promoter and transfected into Oli-neu cells, together with either pcDNA3 (empty vector) or Myrf cloned in pcDNA3. The reporter activity of Sox10-E1 went up substantially in response to Myrf. This increase was abolished when the Myrf motif incidence was mutated. Of note, the Myrf ChIP-seq data show that Myrf does not bind to Sox10-E2 (Fig 3). Consistently, Myrf overexpression did not elevate the reporter activity of pGL3-promoter cloned with Sox10-E2. Shown are data points and their mean and standard error. *p < 7.61 × 10^-3^ by Student’s t test. (D) Mouse OPCs transfected with various reporters, including the wild type and the mutant Sox10-E1, were cultured in the following conditions: 1 day of proliferation (1P), 2 days of differentiation (2D), and 4 days of differentiation (4D). For the mutant Sox10-E1, the Myrf motif incidence was mutated as shown in panel B. Luciferase assay was performed to determine the activity of each reporter, which was normalized by that of pGL3-promoter (the empty vector). Two-way ANOVA revealed that the mutation of the Myrf motif incidence lowers the reporter activity of Sox10-E1 in all three conditions (*p < 5.62 × 10^-5^). The Myrf motif did not interact with the culture condition (*F* > 0.50 and *p* > 0.61). These results do not support the hypothesis that endogenous Myrf acts on the Myrf motif incidence of Sox10-E1. The raw data for S4 Fig are available in S6 Data.
(PDF)

S1 Table The sequences of the gRNAs used for the study. (DOCX)

S1 Data The raw data for Fig 4. (XLS)

S2 Data The raw data for Fig 5B. (XLS)

S3 Data The raw data for Fig 6. (XLS)

S4 Data The raw data for Fig 8A. (XLS)

S5 Data The raw data for S2 Fig. (XLS)

S6 Data The raw data for S4 Fig. (XLSX)

## References

[1] Aggarwal S, Yurlova L, Simons M. Central nervous system myelin: structure, synthesis and assembly. Trends Cell Biol. 2011;21(10):585–93. doi: 10.1016/j.tcb.2011.06.004 21763137

[2] Cohen CCH, Popovic MA, Klooster J, Weil M-T, Möbius W, Nave K-A, et al. Saltatory Conduction along Myelinated Axons Involves a Periaxonal Nanocircuit. Cell. 2020;180(2):311-322.e15. doi: 10.1016/j.cell.2019.11.039 31883793 PMC6978798

[3] Fünfschilling U, Supplie LM, Mahad D, Boretius S, Saab AS, Edgar J, et al. Glycolytic oligodendrocytes maintain myelin and long-term axonal integrity. Nature. 2012;485(7399):517–21. doi: 10.1038/nature11007 22622581 PMC3613737

[4] Lee Y, Morrison BM, Li Y, Lengacher S, Farah MH, Hoffman PN, et al. Oligodendroglia metabolically support axons and contribute to neurodegeneration. Nature. 2012;487(7408):443–8. doi: 10.1038/nature11314 22801498 PMC3408792

[5] Saab AS, Tzvetavona ID, Trevisiol A, Baltan S, Dibaj P, Kusch K, et al. Oligodendroglial NMDA Receptors Regulate Glucose Import and Axonal Energy Metabolism. Neuron. 2016;91(1):119–32. doi: 10.1016/j.neuron.2016.05.016 27292539 PMC9084537

[6] Gibson EM, Purger D, Mount CW, Goldstein AK, Lin GL, Wood LS, et al. Neuronal activity promotes oligodendrogenesis and adaptive myelination in the mammalian brain. Science. 2014;344(6183):1252304. doi: 10.1126/science.1252304 24727982 PMC4096908

[7] McKenzie IA, Ohayon D, Li H, de Faria JP, Emery B, Tohyama K, et al. Motor skill learning requires active central myelination. Science. 2014;346(6207):318–22. doi: 10.1126/science.1254960 25324381 PMC6324726

[8] Steadman PE, Xia F, Ahmed M, Mocle AJ, Penning ARA, Geraghty AC, et al. Disruption of Oligodendrogenesis Impairs Memory Consolidation in Adult Mice. Neuron. 2020;105(1):150-164.e6. doi: 10.1016/j.neuron.2019.10.013 31753579 PMC7579726

[9] Pan S, Mayoral SR, Choi HS, Chan JR, Kheirbek MA. Preservation of a remote fear memory requires new myelin formation. Nat Neurosci. 2020;23(4):487–99. doi: 10.1038/s41593-019-0582-1 32042175 PMC7213814

[10] Wang F, Ren SY, Chen JF, Liu K, Li RX, Li ZF. Myelin degeneration and diminished myelin renewal contribute to age-related deficits in memory. Nat Neurosci. 2020;23(4):481–6. 32042174 10.1038/s41593-020-0588-8 PMC7306053

[11] Wang F, Yang Y-J, Yang N, Chen X-J, Huang N-X, Zhang J, et al. Enhancing Oligodendrocyte Myelination Rescues Synaptic Loss and Improves Functional Recovery after Chronic Hypoxia. Neuron. 2018;99(4):689-701.e5. doi: 10.1016/j.neuron.2018.07.017 30078577 PMC6170028

[12] Makinodan M, Rosen KM, Ito S, Corfas G. A critical period for social experience-dependent oligodendrocyte maturation and myelination. Science. 2012;337(6100):1357–60. doi: 10.1126/science.1220845 22984073 PMC4165613

[13] Liu J, Dietz K, DeLoyht JM, Pedre X, Kelkar D, Kaur J, et al. Impaired adult myelination in the prefrontal cortex of socially isolated mice. Nat Neurosci. 2012;15(12):1621–3. doi: 10.1038/nn.3263 23143512 PMC3729624

[14] Liu J, Dupree JL, Gacias M, Frawley R, Sikder T, Naik P, et al. Clemastine Enhances Myelination in the Prefrontal Cortex and Rescues Behavioral Changes in Socially Isolated Mice. J Neurosci. 2016;36(3):957–62. doi: 10.1523/JNEUROSCI.3608-15.2016 26791223 PMC4719024

[15] Stolt CC, Rehberg S, Ader M, Lommes P, Riethmacher D, Schachner M, et al. Terminal differentiation of myelin-forming oligodendrocytes depends on the transcription factor Sox10. Genes Dev. 2002;16(2):165–70. doi: 10.1101/gad.215802 11799060 PMC155320

[16] Hornig J, Fröb F, Vogl MR, Hermans-Borgmeyer I, Tamm ER, Wegner M. The transcription factors Sox10 and Myrf define an essential regulatory network module in differentiating oligodendrocytes. PLoS Genet. 2013;9(10):e1003907. doi: 10.1371/journal.pgen.1003907 24204311 PMC3814293

[17] Werner T, Hammer A, Wahlbuhl M, Bösl MR, Wegner M. Multiple conserved regulatory elements with overlapping functions determine Sox10 expression in mouse embryogenesis. Nucleic Acids Res. 2007;35(19):6526–38. doi: 10.1093/nar/gkm727 17897962 PMC2095789

[18] Antonellis A, Huynh JL, Lee-Lin S-Q, Vinton RM, Renaud G, Loftus SK, et al. Identification of neural crest and glial enhancers at the mouse Sox10 locus through transgenesis in zebrafish. PLoS Genet. 2008;4(9):e1000174. doi: 10.1371/journal.pgen.1000174 18773071 PMC2518861

[19] Buecker C, Wysocka J. Enhancers as information integration hubs in development: lessons from genomics. Trends Genet. 2012;28(6):276–84. doi: 10.1016/j.tig.2012.02.008 22487374 PMC5064438

[20] Shlyueva D, Stampfel G, Stark A. Transcriptional enhancers: from properties to genome-wide predictions. Nat Rev Genet. 2014;15(4):272–86. doi: 10.1038/nrg3682 24614317

[21] Gasperini M, Hill AJ, McFaline-Figueroa JL, Martin B, Kim S, Zhang MD, et al. A Genome-wide Framework for Mapping Gene Regulation via Cellular Genetic Screens. Cell. 2019;176(1–2):377–390.e19. doi: 10.1016/j.cell.2018.11.029 30612741 PMC6690346

[22] Fulco CP, Munschauer M, Anyoha R, Munson G, Grossman SR, Perez EM, et al. Systematic mapping of functional enhancer-promoter connections with CRISPR interference. Science. 2016;354(6313):769–73. doi: 10.1126/science.aag2445 27708057 PMC5438575

[23] Küspert M, Hammer A, Bösl MR, Wegner M. Olig2 regulates Sox10 expression in oligodendrocyte precursors through an evolutionary conserved distal enhancer. Nucleic Acids Res. 2011;39(4):1280–93. doi: 10.1093/nar/gkq951 20959288 PMC3045606

[24] Dixon JR, Selvaraj S, Yue F, Kim A, Li Y, Shen Y, et al. Topological domains in mammalian genomes identified by analysis of chromatin interactions. Nature. 2012;485(7398):376–80. doi: 10.1038/nature11082 22495300 PMC3356448

[25] Beagan JA, Phillips-Cremins JE. On the existence and functionality of topologically associating domains. Nat Genet. 2020;52(1):8–16. doi: 10.1038/s41588-019-0561-1 31925403 PMC7567612

[26] Creyghton MP, Cheng AW, Welstead GG, Kooistra T, Carey BW, Steine EJ, et al. Histone H3K27ac separates active from poised enhancers and predicts developmental state. Proc Natl Acad Sci U S A. 2010;107(50):21931–6. doi: 10.1073/pnas.1016071107 21106759 PMC3003124

[27] Gilbert LA, Horlbeck MA, Adamson B, Villalta JE, Chen Y, Whitehead EH, et al. Genome-Scale CRISPR-Mediated Control of Gene Repression and Activation. Cell. 2014;159(3):647–61. doi: 10.1016/j.cell.2014.09.029 25307932 PMC4253859

[28] Kearns NA, Pham H, Tabak B, Genga RM, Silverstein NJ, Garber M, et al. Functional annotation of native enhancers with a Cas9-histone demethylase fusion. Nat Methods. 2015;12(5):401–3. doi: 10.1038/nmeth.3325 25775043 PMC4414811

[29] Thakore PI, D’Ippolito AM, Song L, Safi A, Shivakumar NK, Kabadi AM. Highly specific epigenome editing by CRISPR-Cas9 repressors for silencing of distal regulatory elements. Nat Methods. 2015;12(12):1143–9. 26501517 10.1038/nmeth.3630 PMC4666778

[30] Kim D, An H, Shearer RS, Sharif M, Fan C, Choi J-O, et al. A principled strategy for mapping enhancers to genes. Sci Rep. 2019;9(1):11043. doi: 10.1038/s41598-019-47521-w 31363138 PMC6667464

[31] Kim D, Park Y. Molecular mechanism for the multiple sclerosis risk variant rs17594362. Hum Mol Genet. 2019;28(21):3600–9. doi: 10.1093/hmg/ddz216 31509193 PMC6927461

[32] Kim D, An H, Fan C, Park Y. Identifying oligodendrocyte enhancers governing Plp1 expression. Hum Mol Genet. 2021;30(23):2225–39. doi: 10.1093/hmg/ddab184 34230963 PMC8600034

[33] Fan C, Kim D, An H, Park Y. Identifying an oligodendrocyte enhancer that regulates Olig2 expression. Hum Mol Genet. 2023;32(5):835–46. doi: 10.1093/hmg/ddac249 36193754 PMC9941837

[34] Fan C, An H, Kim D, Park Y. Uncovering oligodendrocyte enhancers that control Cnp expression. Hum Mol Genet. 2023;32(23):3225–36. doi: 10.1093/hmg/ddad141 37642363 PMC10656706

[35] Dixon JR, Jung I, Selvaraj S, Shen Y, Antosiewicz-Bourget JE, Lee AY, et al. Chromatin architecture reorganization during stem cell differentiation. Nature. 2015;518(7539):331–6. doi: 10.1038/nature14222 25693564 PMC4515363

[36] Lee D-S, Luo C, Zhou J, Chandran S, Rivkin A, Bartlett A, et al. Simultaneous profiling of 3D genome structure and DNA methylation in single human cells. Nat Methods. 2019;16(10):999–1006. doi: 10.1038/s41592-019-0547-z 31501549 PMC6765423

[37] Deng X, Ma W, Ramani V, Hill A, Yang F, Ay F, et al. Bipartite structure of the inactive mouse X chromosome. Genome Biol. 2015;16(1):152. doi: 10.1186/s13059-015-0728-8 26248554 PMC4539712

[38] Jung M, Krämer E, Grzenkowski M, Tang K, Blakemore W, Aguzzi A, et al. Lines of murine oligodendroglial precursor cells immortalized by an activated neu tyrosine kinase show distinct degrees of interaction with axons in vitro and in vivo. Eur J Neurosci. 1995;7(6):1245–65. doi: 10.1111/j.1460-9568.1995.tb01115.x 7582098

[39] Emery B, Dugas JC. Purification of oligodendrocyte lineage cells from mouse cortices by immunopanning. Cold Spring Harb Protoc. 2013;2013(9):854–68. doi: 10.1101/pdb.prot073973 24003195

[40] Dugas JC, Emery B. Purification of oligodendrocyte precursor cells from rat cortices by immunopanning. Cold Spring Harb Protoc. 2013;2013(8):745–58. doi: 10.1101/pdb.prot070862 23906908

[41] Carpenter AE, Jones TR, Lamprecht MR, Clarke C, Kang IH, Friman O, et al. CellProfiler: image analysis software for identifying and quantifying cell phenotypes. Genome Biol. 2006;7(10):R100. doi: 10.1186/gb-2006-7-10-r100 17076895 PMC1794559

[42] Morabito S, Miyoshi E, Michael N, Shahin S, Martini AC, Head E, et al. Single-nucleus chromatin accessibility and transcriptomic characterization of Alzheimer’s disease. Nat Genet. 2021;53(8):1143–55. doi: 10.1038/s41588-021-00894-z 34239132 PMC8766217

[43] Nott A, Holtman IR, Coufal NG, Schlachetzki JCM, Yu M, Hu R, et al. Brain cell type-specific enhancer-promoter interactome maps and disease-risk association. Science. 2019;366(6469):1134–9. doi: 10.1126/science.aay0793 31727856 PMC7028213

[44] The Roadmap Epigenomics Consortium. Integrative analysis of 111 reference human epigenomes. Nature. 2015;518:317–30. 25693563 10.1038/nature14248 PMC4530010

[45] Cusanovich DA, Hill AJ, Aghamirzaie D, Daza RM, Pliner HA, Berletch JB, et al. A single-cell atlas of in vivo mammalian chromatin accessibility. Cell. 2018;174(5):1309–24. 30078704 10.1016/j.cell.2018.06.052 PMC6158300

[46] Kaufman CK, Mosimann C, Fan ZP, Yang S, Thomas AJ, Ablain J, et al. A zebrafish melanoma model reveals emergence of neural crest identity during melanoma initiation. Science. 2016;351(6272):aad2197. doi: 10.1126/science.aad2197 26823433 PMC4868069

[47] Mauduit D, Taskiran II, Minnoye L, de Waegeneer M, Christiaens V, Hulselmans G, et al. Analysis of long and short enhancers in melanoma cell states. Elife. 2021;10:e71735. doi: 10.7554/eLife.71735 34874265 PMC8691835

[48] Antonellis A, Bennett WR, Menheniott TR, Prasad AB, Lee-Lin S-Q, NISC Comparative Sequencing Program, et al. Deletion of long-range sequences at Sox10 compromises developmental expression in a mouse model of Waardenburg-Shah (WS4) syndrome. Hum Mol Genet. 2006;15(2):259–71. doi: 10.1093/hmg/ddi442 16330480

[49] Bujalka H, Koenning M, Jackson S, Perreau VM, Pope B, Hay CM, et al. MYRF is a membrane-associated transcription factor that autoproteolytically cleaves to directly activate myelin genes. PLoS Biol. 2013;11(8):e1001625. doi: 10.1371/journal.pbio.1001625 23966833 PMC3742440

[50] Kim D, Choi J-o, Fan C, Shearer RS, Sharif M, Busch P, et al. Homo-trimerization is essential for the transcription factor function of Myrf for oligodendrocyte differentiation. Nucleic Acids Research. 2017;45(9):5112–25. 28160598 10.1093/nar/gkx080 PMC5436001

[51] Choi J-O, Fan C, Kim D, Sharif M, An H, Park Y. Elucidating the transactivation domain of the pleiotropic transcription factor Myrf. Sci Rep. 2018;8(1):13075. doi: 10.1038/s41598-018-31477-4 30166609 PMC6117317

[52] Xu W, Yang W, Zhang Y, Chen Y, Hong N, Zhang Q, et al. ISSAAC-seq enables sensitive and flexible multimodal profiling of chromatin accessibility and gene expression in single cells. Nat Methods. 2022;19(10):1243–9. doi: 10.1038/s41592-022-01601-4 36109677

[53] Liu Z, Hu X, Cai J, Liu B, Peng X, Wegner M, et al. Induction of oligodendrocyte differentiation by Olig2 and Sox10: evidence for reciprocal interactions and dosage-dependent mechanisms. Dev Biol. 2007;302(2):683–93. doi: 10.1016/j.ydbio.2006.10.007 17098222

[54] Cossais F, Wahlbuhl M, Kriesch J, Wegner M. SOX10 structure-function analysis in the chicken neural tube reveals important insights into its role in human neurocristopathies. Hum Mol Genet. 2010;19(12):2409–20. doi: 10.1093/hmg/ddq124 20308050

[55] Bondurand N, Fouquet V, Baral V, Lecerf L, Loundon N, Goossens M, et al. Alu-mediated deletion of SOX10 regulatory elements in Waardenburg syndrome type 4. Eur J Hum Genet. 2012;20(9):990–4. doi: 10.1038/ejhg.2012.29 22378281 PMC3421117

[56] Lecerf L, Kavo A, Ruiz-Ferrer M, Baral V, Watanabe Y, Chaoui A, et al. An impairment of long distance SOX10 regulatory elements underlies isolated Hirschsprung disease. Hum Mutat. 2014;35(3):303–7. doi: 10.1002/humu.22499 24357527

[57] Huang Y, Geng J, Long Y, Xiong W, Kang L, Chen M. Five novel cis-regulatory deletions of SOX10 cause Waardenburg syndrome type II. Frontiers in Audiology and Otology. 2024;2.

[58] Flavahan WA, Drier Y, Liau BB, Gillespie SM, Venteicher AS, Stemmer-Rachamimov AO, et al. Insulator dysfunction and oncogene activation in IDH mutant gliomas. Nature. 2016;529(7584):110–4. doi: 10.1038/nature16490 26700815 PMC4831574

[59] Gong Y, Lazaris C, Sakellaropoulos T, Lozano A, Kambadur P, Ntziachristos P, et al. Stratification of TAD boundaries reveals preferential insulation of super-enhancers by strong boundaries. Nat Commun. 2018;9(1):542. doi: 10.1038/s41467-018-03017-1 29416042 PMC5803259

[60] Emery B, Agalliu D, Cahoy JD, Watkins TA, Dugas JC, Mulinyawe SB, et al. Myelin gene regulatory factor is a critical transcriptional regulator required for CNS myelination. Cell. 2009;138(1):172–85. doi: 10.1016/j.cell.2009.04.031 19596243 PMC2757090

[61] Grant CE, Bailey TL, Noble WS. FIMO: scanning for occurrences of a given motif. Bioinformatics. 2011;27(7):1017–8. 21330290 10.1093/bioinformatics/btr064 PMC3065696

[62] Mátés L, Chuah MKL, Belay E, Jerchow B, Manoj N, Acosta-Sanchez A, et al. Molecular evolution of a novel hyperactive Sleeping Beauty transposase enables robust stable gene transfer in vertebrates. Nat Genet. 2009;41(6):753–61. doi: 10.1038/ng.343 19412179

[63] Yusa K, Zhou L, Li MA, Bradley A, Craig NL. A hyperactive piggyBac transposase for mammalian applications. Proc Natl Acad Sci U S A. 2011;108(4):1531–6. doi: 10.1073/pnas.1008322108 21205896 PMC3029773

[64] Breunig JJ, Levy R, Antonuk CD, Molina J, Dutra-Clarke M, Park H, et al. Ets Factors Regulate Neural Stem Cell Depletion and Gliogenesis in Ras Pathway Glioma. Cell Rep. 2015;12(2):258–71. doi: 10.1016/j.celrep.2015.06.012 26146073

[65] Fan C, An H, Sharif M, Kim D, Park Y. Functional mechanisms of MYRF DNA-binding domain mutations implicated in birth defects. J Biol Chem. 2021;296:100612. doi: 10.1016/j.jbc.2021.100612 33798553 PMC8094900

[66] Yu Y, Chen Y, Kim B, Wang H, Zhao C, He X, et al. Olig2 targets chromatin remodelers to enhancers to initiate oligodendrocyte differentiation. Cell. 2013;152(1–2):248–61. doi: 10.1016/j.cell.2012.12.006 23332759 PMC3553550

[67] He D, Marie C, Zhao C, Kim B, Wang J, Deng Y, et al. Chd7 cooperates with Sox10 and regulates the onset of CNS myelination and remyelination. Nat Neurosci. 2016;19(5):678–89. doi: 10.1038/nn.4258 26928066 PMC4846514

[68] Liu Z, Yan M, Liang Y, Liu M, Zhang K, Shao D, et al. Nucleoporin Seh1 Interacts with Olig2/Brd7 to Promote Oligodendrocyte Differentiation and Myelination. Neuron. 2019;102(3):587-601.e7. doi: 10.1016/j.neuron.2019.02.018 30876848 PMC6508993

[69] Zhang L, He X, Liu L, Jiang M, Zhao C, Wang H, et al. Hdac3 Interaction with p300 Histone Acetyltransferase Regulates the Oligodendrocyte and Astrocyte Lineage Fate Switch. Dev Cell. 2016;36(3):316–30. doi: 10.1016/j.devcel.2016.01.002 26859354 PMC4750051

[70] He D, Wang J, Lu Y, Deng Y, Zhao C, Xu L, et al. lncRNA Functional Networks in Oligodendrocytes Reveal Stage-Specific Myelination Control by an lncOL1/Suz12 Complex in the CNS. Neuron. 2017;93(2):362–78. doi: 10.1016/j.neuron.2016.11.044 28041882 PMC5600615

[71] Zhao C, Deng Y, Liu L, Yu K, Zhang L, Wang H, et al. Dual regulatory switch through interactions of Tcf7l2/Tcf4 with stage-specific partners propels oligodendroglial maturation. Nat Commun. 2016;7:10883. doi: 10.1038/ncomms10883 26955760 PMC4786870

[72] Ou Z, Sun Y, Lin L, You N, Liu X, Li H, et al. Olig2-Targeted G-Protein-Coupled Receptor Gpr17 Regulates Oligodendrocyte Survival in Response to Lysolecithin-Induced Demyelination. J Neurosci. 2016;36(41):10560–73. doi: 10.1523/JNEUROSCI.0898-16.2016 27733608 PMC6601930

[73] Lopez-Anido C, Sun G, Koenning M, Srinivasan R, Hung HA, Emery B, et al. Differential Sox10 genomic occupancy in myelinating glia. Glia. 2015;63(11):1897–914. doi: 10.1002/glia.22855 25974668 PMC4644515

[74] Zhao C, Dong C, Frah M, Deng Y, Marie C, Zhang F, et al. Dual Requirement of CHD8 for Chromatin Landscape Establishment and Histone Methyltransferase Recruitment to Promote CNS Myelination and Repair. Dev Cell. 2018;45(6):753-768.e8. doi: 10.1016/j.devcel.2018.05.022 29920279 PMC6063525

[75] Elbaz B, Aaker JD, Isaac S, Kolarzyk A, Brugarolas P, Eden A, et al. Phosphorylation State of ZFP24 Controls Oligodendrocyte Differentiation. Cell Rep. 2018;23(8):2254–63. doi: 10.1016/j.celrep.2018.04.089 29791837 PMC6002757

[76] Laitman BM, Asp L, Mariani JN, Zhang J, Liu J, Sawai S, et al. The Transcriptional Activator Krüppel-like Factor-6 Is Required for CNS Myelination. PLoS Biol. 2016;14(5):e1002467. doi: 10.1371/journal.pbio.1002467 27213272 PMC4877075

[77] Liu J, Magri L, Zhang F, Marsh NO, Albrecht S, Huynh JL, et al. Chromatin landscape defined by repressive histone methylation during oligodendrocyte differentiation. J Neurosci. 2015;35(1):352–65. doi: 10.1523/JNEUROSCI.2606-14.2015 25568127 PMC4287153

[78] Langmead B, Salzberg SL. Fast gapped-read alignment with Bowtie 2. Nat Methods. 2012;9(4):357–9. doi: 10.1038/nmeth.1923 22388286 PMC3322381

[79] Zhang Y, Liu T, Meyer CA, Eeckhoute J, Johnson DS, Bernstein BE, et al. Model-based analysis of ChIP-Seq (MACS). Genome Biol. 2008;9(9):R137. doi: 10.1186/gb-2008-9-9-r137 18798982 PMC2592715

[80] Dobin A, Davis CA, Schlesinger F, Drenkow J, Zaleski C, Jha S, et al. STAR: ultrafast universal RNA-seq aligner. Bioinformatics. 2013;29(1):15–21. doi: 10.1093/bioinformatics/bts635 23104886 PMC3530905

[81] Hao Y, Hao S, Andersen-Nissen E, Mauck WM 3rd, Zheng S, Butler A, et al. Integrated analysis of multimodal single-cell data. Cell. 2021;184(13):3573-3587.e29. doi: 10.1016/j.cell.2021.04.048 34062119 PMC8238499

[82] Stuart T, Srivastava A, Madad S, Lareau CA, Satija R. Single-cell chromatin state analysis with Signac. Nat Methods. 2021;18(11):1333–41. doi: 10.1038/s41592-021-01282-5 34725479 PMC9255697

[83] Trapnell C, Cacchiarelli D, Grimsby J, Pokharel P, Li S, Morse M, et al. The dynamics and regulators of cell fate decisions are revealed by pseudotemporal ordering of single cells. Nat Biotechnol. 2014;32(4):381–6. doi: 10.1038/nbt.2859 24658644 PMC4122333

[84] Kerpedjiev P, Abdennur N, Lekschas F, McCallum C, Dinkla K, Strobelt H, et al. HiGlass: web-based visual exploration and analysis of genome interaction maps. Genome Biol. 2018;19(1):125. doi: 10.1186/s13059-018-1486-1 30143029 PMC6109259

[85] Robinson JT, Thorvaldsdóttir H, Winckler W, Guttman M, Lander ES, Getz G, et al. Integrative genomics viewer. Nat Biotechnol. 2011;29(1):24–6. doi: 10.1038/nbt.1754 21221095 PMC3346182

