# Supplementary figures and images for: Discovery of oligodendrocyte enhancers that regulate Sox10 expression
Source: PLoS Genet. 2025 Jul 11;21(7):e1011778. doi: 10.1371/journal.pgen.1011778 (PMC12266436; doi:10.1371/journal.pgen.1011778)

**S1 Fig. Transposon-based plasmids used to generate stable cell lines**

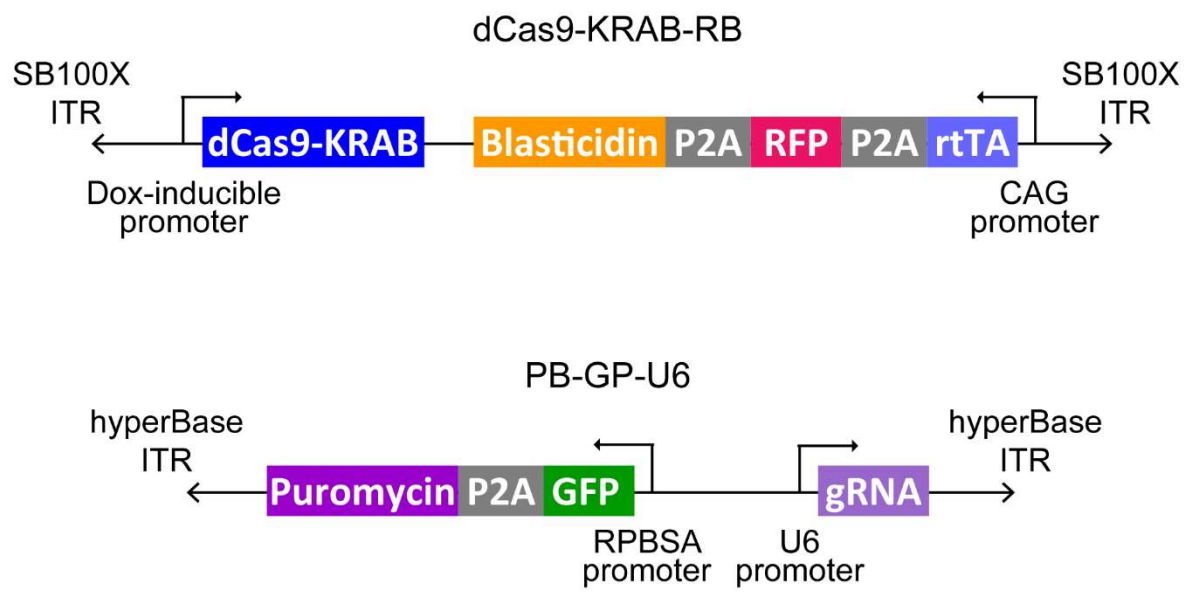

Supplement: S1 Fig — (PDF) [file pgen.1011778.s001.pdf]

**S3 Fig. The NIH Roadmap Epigenomics Project H3K27ac ChIP-seq data for Sox10-E1 and Sox10-E2**

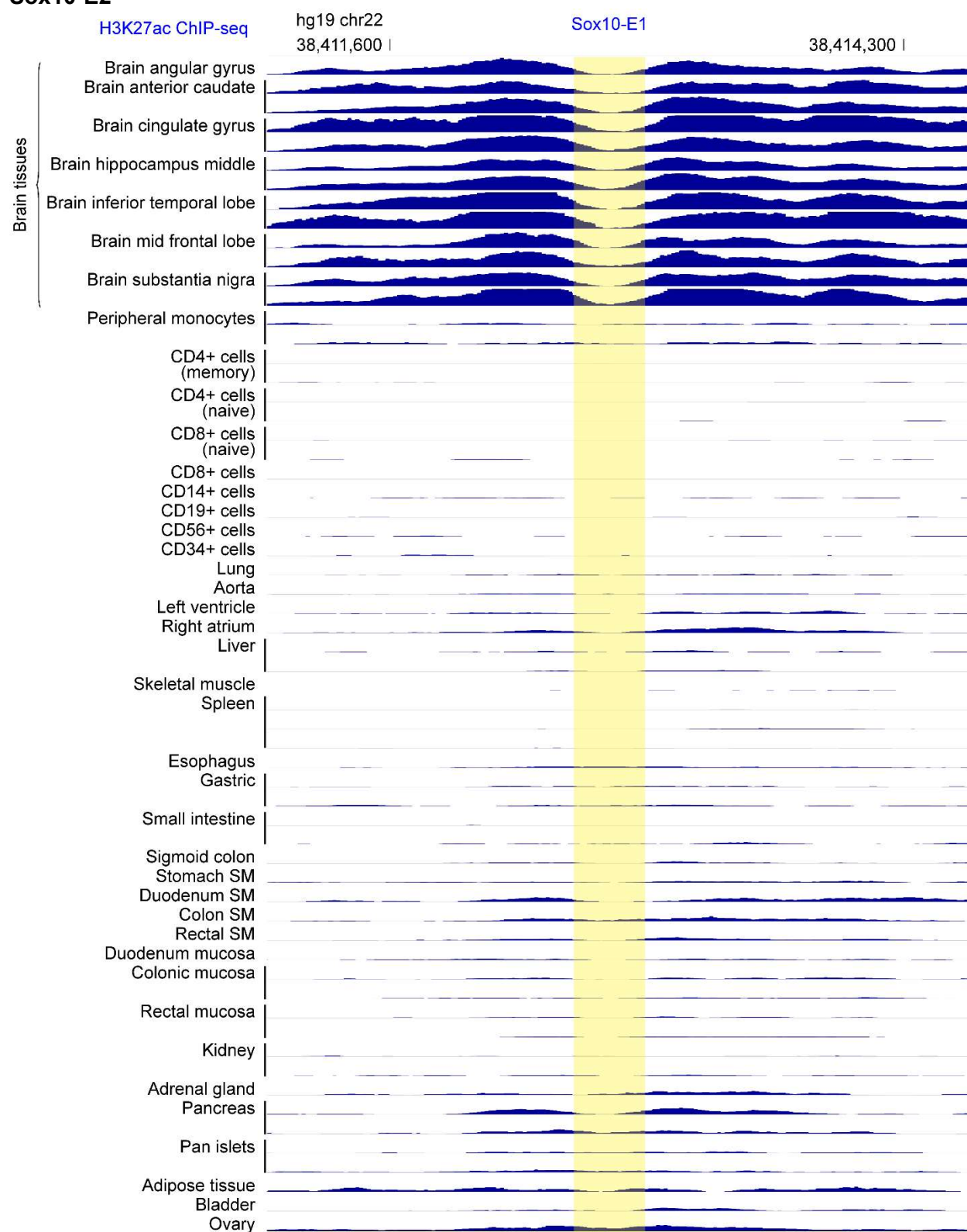

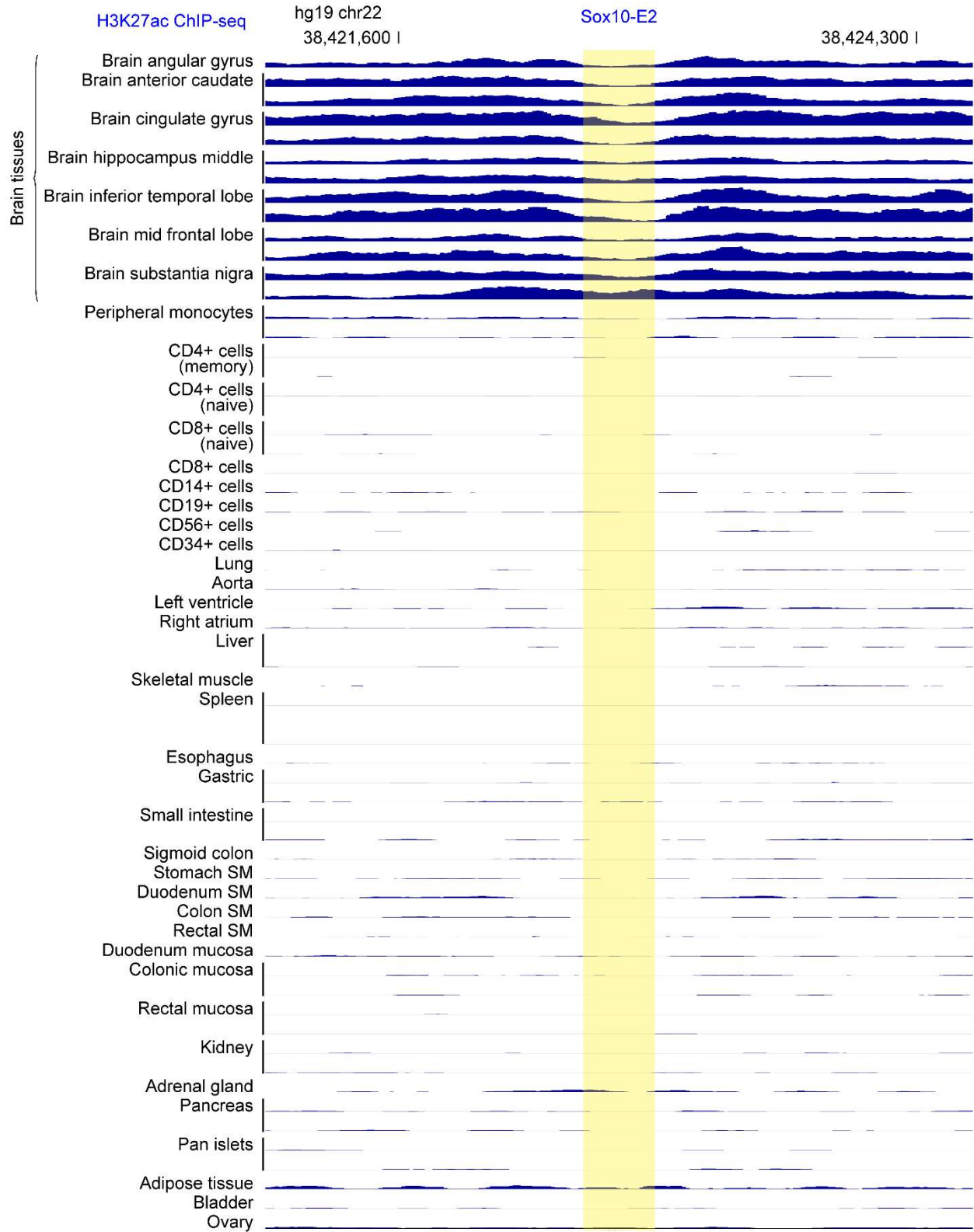

Supplement: S3 Fig — (PDF) [file pgen.1011778.s003.pdf]
