# Supplementary material for: Discovery of oligodendrocyte enhancers that regulate Sox10 expression
Source: PLoS Genet. 2025 Jul 11;21(7):e1011778. doi: 10.1371/journal.pgen.1011778 (PMC12266436; doi:10.1371/journal.pgen.1011778)
Supplement: S2 Fig — (A) dCas9-KRAB was delivered to NC1, a region 1 kb upstream of the Sox10 promoter, by 8 different gRNAs. The expression level of Sox10 was measured by a luciferase assay with ECR9. (Left) The locations of the 8 gRNAs. (Right) The luciferase assay results. Pro2 was used as a positive control. Shown are data points and their mean and standard error. *p < 6.91 × 10^-4^ by Student’s t test. None of the 8 gRNAs affected the transcription of Sox10. (B) dCas9-KRAB was delivered to NC2, a region between the Sox10 promoter and EC1, by 12 different gRNAs. The expression level of Sox10 was measured by a luciferase assay with ECR9. (Left) The locations of the 12 gRNAs. G6, which is between G1 and G4, is not shown because of a low BLAT mapping score. (Right) The luciferase assay results. Pro2 was used as a positive control. Shown are data points and their mean and standard error. *p < 2.03 × 10^-3^ by Student’s t test. None of the 12 gRNAs affected the transcription of Sox10. (C) dCas9-KRAB was delivered to NC3, a region between EC1 and EC2, by 12 different gRNAs. The expression level of Sox10 was measured by a luciferase assay with ECR9. (Left) The locations of the 12 gRNAs. G9, which is between G6 and G7, is not shown because of a low BLAT mapping score. (Right) The luciferase assay results. Pro2 was used as a positive control. Shown are data points and their mean and standard error. *p < 9.77 × 10^-3^ by Student’s t test. Promoter contact is not a privilege limited to enhancers. Non-enhancer regions can also contact the promoter of a gene. Though rare, some loci between EC1 and EC2 may contact the Sox10 promoter and be open enough. If dCas9-KRAB is targeted at such a locus, the expression of Sox10 would be downregulated, even though the targeted locus is not an enhancer. This phenomenon has previously been reported for a different gene (Science 2016 354:769). Of the 12 gRNAs, 3 led to the downregulation of Sox10. Notably, all three gRNAs map to the 3’ end of the region, arguing aga [file pgen.1011778.s002.pdf]

S2 Fig. CRISPRi analysis of three control regions

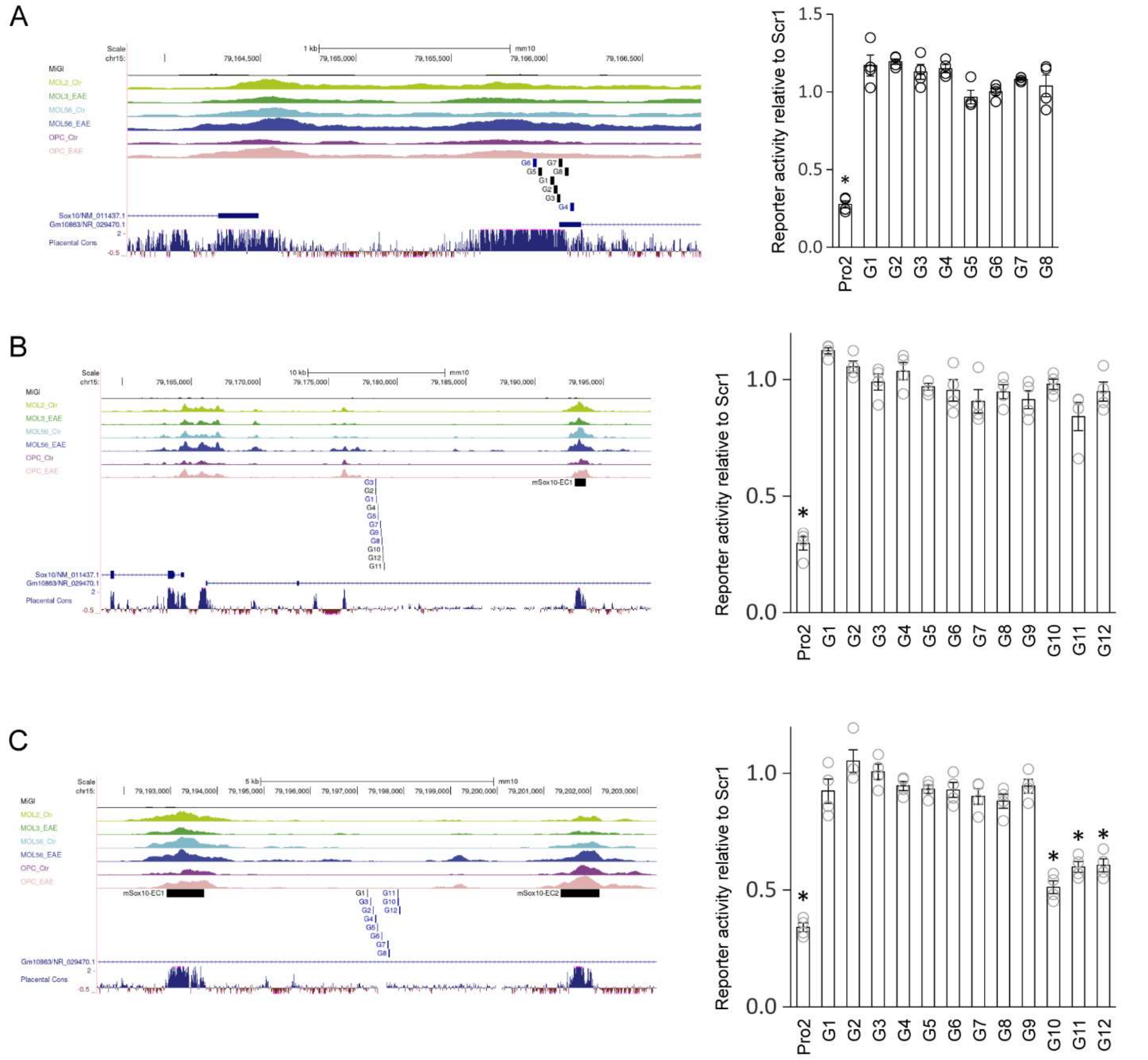

(A) dCas9-KRAB was delivered to NC1, a region 1 kb upstream of the *Sox10* promoter, by 8 different gRNAs. The expression level of *Sox10* was measured by a luciferase assay with ECR9. (Left) The locations of the 8 gRNAs. (Right) The luciferase assay results. Pro2 was used as a positive control. Shown are data points and their mean and standard error. *$p < 6.91 \times 10^{-4}$ by Student's *t* test. None of the 8 gRNAs affected the transcription of *Sox10*.

(B) dCas9-KRAB was delivered to NC2, a region between the *Sox10* promoter and EC1, by 12 different gRNAs. The expression level of *Sox10* was measured by a luciferase assay with ECR9. (Left) The locations of the 12 gRNAs. G6, which is between G1 and G4, is not shown because of a low BLAT mapping score. (Right) The luciferase assay results. Pro2 was used as a positive control. Shown are data points and their mean and standard error. *$p < 2.03 \times 10^{-3}$ by Student's *t* test. None of the 12 gRNAs affected the transcription of *Sox10*.

(C) dCas9-KRAB was delivered to NC3, a region between EC1 and EC2, by 12 different gRNAs. The expression level of *Sox10* was measured by a luciferase assay with ECR9. (Left) The locations of the 12 gRNAs. G9, which is between G6 and G7, is not shown because of a low BLAT mapping score. (Right) The luciferase assay results. Pro2 was used as a positive control. Shown are data points and their mean and standard error. $^*p < 9.77\times10^{-3}$ by Student's *t* test. Promoter contact is not a privilege limited to enhancers. Non-enhancer regions can also contact the promoter of a gene. Though rare, some loci between EC1 and EC2 may contact the *Sox10* promoter and be open enough. If dCas9-KRAB is targeted at such a locus, the expression of *Sox10* would be downregulated, even though the targeted locus is not an enhancer. This phenomenon has previously been reported for a different gene (*Science* 2016 354:769). Of the 12 gRNAs, 3 led to the downregulation of *Sox10*. Notably, all three gRNAs map to the 3' end of the region, arguing against the idea that CRISPRi repression non-specifically spreads from the target site to the *Sox10* promoter. Rather, these results suggest that the 3' end of the region contacts the *Sox10* promoter and is open enough.
