# Supplementary material for: Discovery of oligodendrocyte enhancers that regulate Sox10 expression
Source: PLoS Genet. 2025 Jul 11;21(7):e1011778. doi: 10.1371/journal.pgen.1011778 (PMC12266436; doi:10.1371/journal.pgen.1011778)
Supplement: S4 Fig — (A) The Myrf motif that mediates the sequence-specific DNA binding of Myrf. (B) DNA pulldown assay for Myrf and Sox10-E1. FLAG-Myrf (Myrf with an N-terminal FLAG tag) was expressed in Oli-neu cells, and cell lysates were mixed with either bare beads or beads coated with duplex DNA oligos (Sox10-E1-WT and Sox10-E1-MU). The duplex oligos contained either the wild type motif incidence (Sox10-E1-WT) or a mutated one (Sox10-E1-MU). The mixtures were separated into the sup and bead fractions by centrifuge, and both fractions were probed by FLAG antibodies. Immunoblotting showed that Myrf specifically bound Sox10-E1-WT and that this binding became weaker when the motif incidence was mutated (the bead fractions). The sup fraction revealed that comparable amounts of proteins were used for the three binding reactions, ruling out the trivial possibility that the specific binding of Myrf to Sox10-E1-WT is due to unequal protein amounts used for the binding reactions. IB: immunoblotting. (C) To test the functional significance of the Myrf motif incidence in Sox10-E1, we performed a luciferase assay in Oli-neu cells. The wild type and the mutant versions of Sox10-E1 were cloned into pGL3-promoter and transfected into Oli-neu cells, together with either pcDNA3 (empty vector) or Myrf cloned in pcDNA3. The reporter activity of Sox10-E1 went up substantially in response to Myrf. This increase was abolished when the Myrf motif incidence was mutated. Of note, the Myrf ChIP-seq data show that Myrf does not bind to Sox10-E2 (Fig 3). Consistently, Myrf overexpression did not elevate the reporter activity of pGL3-promoter cloned with Sox10-E2. Shown are data points and their mean and standard error. *p < 7.61 × 10^-3^ by Student’s t test. (D) Mouse OPCs transfected with various reporters, including the wild type and the mutant Sox10-E1, were cultured in the following conditions: 1 day of proliferation (1P), 2 days of differentiation (2D), and 4 days of differentiation (4D). For the mutant So [file pgen.1011778.s004.pdf]

S4 Fig. Overexpressed Myrf, but not endogenous Myrf, acts on Sox10-E1

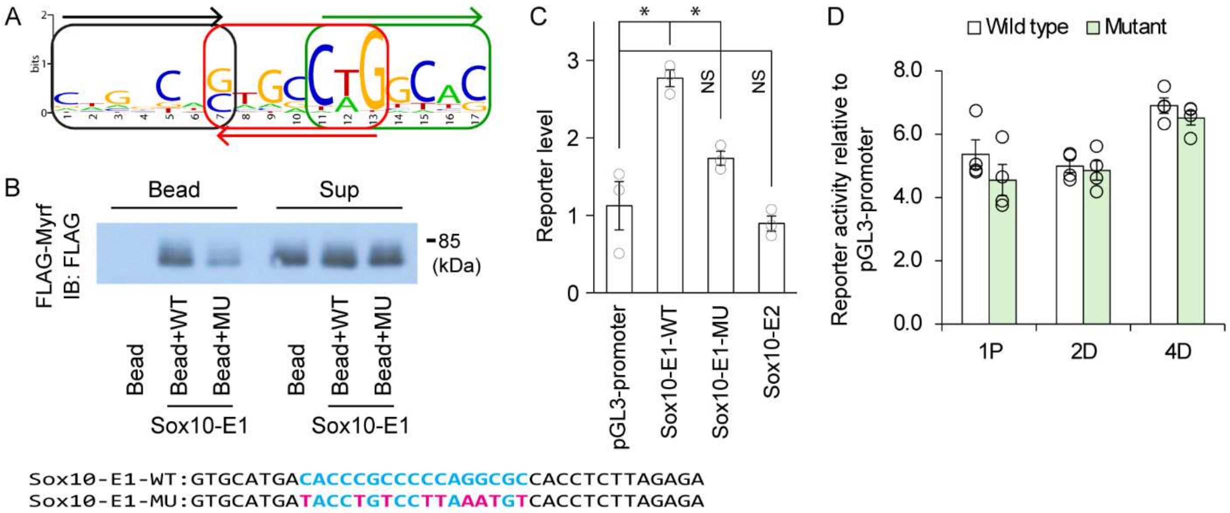

(A) The Myrf motif that mediates the sequence-specific DNA binding of Myrf. (B) DNA pulldown assay for Myrf and Sox10-E1. FLAG-Myrf (Myrf with an N-terminal FLAG tag) was expressed in Oli-neu cells, and cell lysates were mixed with either bare beads or beads coated with duplex DNA oligos (Sox10-E1-WT and Sox10-E1-MU). The duplex oligos contained either the wild type motif incidence (Sox10-E1-WT) or a mutated one (Sox10-E1-MU). The mixtures were separated into the sup and bead fractions by centrifuge, and both fractions were probed by FLAG antibodies. Immunoblotting showed that Myrf specifically bound Sox10-E1-WT and that this binding became weaker when the motif incidence was mutated (the bead fractions). The sup fraction revealed that comparable amounts of proteins were used for the three binding reactions, ruling out the trivial possibility that the specific binding of Myrf to Sox10-E1-WT is due to unequal protein amounts used for the binding reactions. IB: immunoblotting. (C) To test the functional significance of the Myrf motif incidence in Sox10-E1, we performed a luciferase assay in Oli-neu cells. The wild type and the mutant versions of Sox10-E1 were cloned into pGL3-promoter and transfected into Oli-neu cells, together with either pcDNA3 (empty vector) or Myrf cloned in pcDNA3. The reporter activity of Sox10-E1 went up substantially in response to Myrf. This increase was abolished when the Myrf motif incidence was mutated. Of note, the Myrf ChIP-seq data show that Myrf does not bind to Sox10-E2 (**Figure 3**). Consistently, Myrf overexpression did not elevate the reporter activity of pGL3-promoter cloned with Sox10-E2. Shown are data points and their mean and standard error. *$p < 7.61\times10^{-3}$ by Student's t test. (D) Mouse OPCs transfected with various reporters, including the wild type and the mutant Sox10-E1, were cultured in the following conditions: 1 day of proliferation (1P), 2 days of differentiation (2D), and 4 days of differentiation (4D). For the mutant Sox10-E1, the Myrf motif incidence was mutated as shown in panel B. Luciferase assay was performed to determine the activity of each reporter, which was normalized by that of pGL3-promoter (the empty vector). Two-way ANOVA revealed that the mutation of the Myrf motif incidence lowers the reporter activity of Sox10-E1 in all three conditions (*$p < 5.62\times10^{-5}$). The Myrf motif did not interact with the culture condition ($F > 0.50$ and $p > 0.61$). These results do not support the hypothesis that endogenous Myrf acts on the Myrf motif incidence of Sox10-E1.
