# Supplementary material for: Discovery of oligodendrocyte enhancers that regulate Sox10 expression
Source: PLoS Genet. 2025 Jul 11;21(7):e1011778. doi: 10.1371/journal.pgen.1011778 (PMC12266436; doi:10.1371/journal.pgen.1011778)
Supplement: S1 Table — (DOCX) [file pgen.1011778.s005.docx]

**S1 Table. The sequences of the gRNAs used for the study**

| Scr1 | GCACTACCAGAGCTAACTCA |
| --- | --- |
| Scr2 | TGCGAATACGCCCACGCGAT |
| Pro1 | GGGGATGAAGGCAGCGCTGG |
| Pro2 | ATCCTCACCACCAAACACCC |
| EC1-G1 | GCTGCTTGGCAGGACTTGGT |
| EC1-G2 | GGTTCCTCTTGTTTATGAGC |
| EC1-G3 | GCACTCTTTCCACCCGCCCA |
| EC1-G4 | AAAGCGCGGGGGGACAATGC |
| EC1-G5 | TGTCTCCGAAGGGTTAACGA |
| EC1-G6 | TGTGCATGACACCCGCCCCC |
| EC1-G7 | AGTATAATACAGGGAGGCGT |
| EC1-G8 | CACCAGAGCTCTTGCTCGGC |
| EC1-G9 | AGCTGCCAGCACATCCGCCC |
| EC1-G10 | TTAGGGTCTGCATTCCGATG |
| EC1-G11 | GTGTCTGAGGTCTGCGGGAT |
| EC1-G12 | CCTCAAGCCCCATGCCCCCC |
| EC2-G1 | GGGCGGCTTTGGGGTGACCC |
| EC2-G2 | TGCAGACTGGGGAGACAATC |
| EC2-G3 | GAAGGGGACCGCAGCTGTAG |
| EC2-G4 | GGCTGTCCCTCTTGGGGGCG |
| EC2-G5 | GTGTGGCCTTGTCAACCTTC |
| EC2-G6 | ACAAAGCAGATAGACTAGTC |
| EC2-G7 | GAAGTGGTACTGTTACCTTC |
| EC2-G8 | TGGAGAGGGTCTTGCGGTTA |
| EC2-G9 | GGGCCCGGCTGGGCAACTGG |
| EC2-G10 | AGCCATTCTCCACGTGGTTG |
| EC2-G11 | CAGGCGACTCATTAGCATTG |
| EC2-G12 | CTGCTGGTGATAAAGCTATC |
| EC3-G1 | GCCTCGCTTGGAAAACAACT |
| EC3-G2 | AAAAGGTCCCTTTGTGGCGC |
| EC3-G3 | CCCTACTCTGCGCGCACCCC |
| EC3-G4 | TCTCTCTCCCAAGGCGCCTT |
| EC3-G5 | CCCTCATGGCTCATCCGGCC |
| EC3-G6 | TCCTTACATCTCCCGGGTTC |
| EC3-G7 | AGACCCTCAACATGGCCGCT |
| EC3-G8 | GCCCCTGTGCCCGCGCCCAT |
| EC3-G9 | GCTGGAGGGGCCTACGGGGC |
| EC3-G10 | GTTTTCAAAATGGCCGTCGC |
| EC3-G11 | AGGAAGGTCCCGTTTCAGCC |
| EC3-G12 | GTCCTGAGAGGTAGAGACCG |
| EC3-G13 | TGTGCAGGTTAAGATGGTTG |
| EC4-G1 | CTGAAATGCCGCCTTTGTGC |
| EC4-G2 | CTGATACTGAGCGGCCCAGC |
| EC4-G3 | GAGGGGGAGTTAACCCTTAG |
| EC4-G4 | TGGGCAGCCCCTCGTTGCTC |
| EC4-G5 | TAGACGTGCCTTACCATCCC |
| EC4-G6 | GCCATGGGCACTGGCATAGT |
| EC4-G7 | TTGAGTGGCCCAGCCTGACC |
| EC4-G8 | GGGATTGGGCACCCTGGTTA |
| EC4-G9 | CTCTGGATAAGGGTTAATTC |
| EC4-G10 | ATGGTGGGGCCCCATAAGAG |
| EC4-G11 | ACAAGATGACCGTAAGCCTT |
| EC4-G12 | TGCACACAACCTGCTAACAC |
| EC5-G1 | GAGACAGCAGAGTCGCAGTC |
| EC5-G2 | CTCTGACCATTCCTCCGGCA |
| EC5-G3 | ATTAATAGCAAGGAGTCGAC |
| EC5-G4 | ATTCCTCTGGGAGCTGATCG |
| EC5-G5 | AAGTTGGCCTGCGCTCACTC |
| EC5-G6 | TTGTCCCTGGGAACCGTGCT |
| EC5-G7 | GTGGTTCACTTGCAAAGGGG |
| EC5-G8 | CCCTGTTCCCCGGTCTACTC |
| EC5-G9 | GATATCAAGAGAAGCGCATG |
| EC5-G10 | TGGGGACATAGATGCATCCT |
| EC5-G11 | GGCTGGACTCTCCATAGGAT |
| EC5-G12 | CTAGGTTCACCTGAACCCCC |
| EC5-G13 | GCTAGGTCAGAGGAATTCCT |
| NC1-G1 | GCTTTTTAACAGCGCCCGCC |
| NC1-G2 | CGCCCGGCCTCAGAACCGCC |
| NC1-G3 | CGCCCGGAGAGAAGCCGCCT |
| NC1-G4 | CAGCAGTTGGAGACCGCTGC |
| NC1-G5 | GCTGTCTTTGTTCGAAATAC |
| NC1-G6 | CAAGGCCCCGAACAAGAGTC |
| NC1-G7 | AAGCCGCCTTGGATGGACAG |
| NC1-G8 | CAGCAAGCATCCGGCCTCCC |
| NC2-G1 | CAATTCCTAGCACCCGCATC |
| NC2-G2 | GTGTATGCTGGCGCAGTCAC |
| NC2-G3 | ACACCCTCCTTTGGCCGCTG |
| NC2-G4 | AAACAGCACCGCCCACGTTC |
| NC2-G5 | TGATCTAGATTAGACCTAGG |
| NC2-G6 | AAGGAAGGAAAGCGAAGGCC |
| NC2-G7 | ACCCAGGTCCATTCGACTCC |
| NC2-G8 | TGTGAACCACGTCACAGAAT |
| NC2-G9 | TCCCACCCTGGCTAGGAATA |
| NC2-G10 | TCGTTAAATAACTGACCCAG |
| NC2-G11 | CACTCCCAAACTCTGTATAG |
| NC2-G12 | GCCTCAAAGGTGGTCAGCAA |
| NC3-G1 | TGTCACATGGACACTCACGC |
| NC3-G2 | TAGAGGTTGCTACTAGCATG |
| NC3-G3 | AATAAGAAGATGGCATCTAA |
| NC3-G4 | TATAGCTTTAGTAAGCGAGC |
| NC3-G5 | AAAGGGTTTCTGAACCTCTC |
| NC3-G6 | ATGAGATCTGACTCCTCTTC |
| NC3-G7 | GAACTGAACAGACCCTAAAG |
| NC3-G8 | ATAAAGAAAAGGATCTGGCT |
| NC3-G9 | AGCACTGACTGCTCTTCTGA |
| NC3-G10 | AAATAGAGAATGCCGGGCAG |
| NC3-G11 | TAGAGAATGCCGGGCAGTGG |
| NC3-G12 | TATTCTGCAAATGTGTAATA |
